# In Vitro CO-Releasing and Antioxidant Properties of Sulfonamide-Based CAI-CORMs in a H_2_O_2_-Stimulated Human Achilles Tendon-Derived Cell Model

**DOI:** 10.3390/molecules30030593

**Published:** 2025-01-28

**Authors:** Emanuela Berrino, Paolo Guglielmi, Fabrizio Carta, Simone Carradori, Cristina Campestre, Andrea Angeli, Francesca Arrighi, Virginia Pontecorvi, Paola Chimenti, Daniela Secci, Claudiu T. Supuran, Marialucia Gallorini

**Affiliations:** 1 Department of Life Science, Health, and Health Professions, Link Campus University, Via del Casale di San Pio V, 44, 00165 Rome, Italy; e.berrino@unilink.it; 2 Department of Drug Chemistry and Technologies, Sapienza University of Rome, P.le A. Moro 5, 00185 Rome, Italy; francesca.arrighi@uniroma1.it (F.A.); virginia.pontecorvi@uniroma1.it (V.P.); paola.chimenti@uniroma1.it (P.C.); daniela.secci@uniroma1.it (D.S.); 3 NEUROFARBA Department, Sezione di Scienze Farmaceutiche e Nutraceutiche, University of Florence, Sesto Fiorentino, 50019 Florence, Italy; fabrizio.carta@unifi.it (F.C.); andrea.angeli@unifi.it (A.A.); claudiu.supuran@unifi.it (C.T.S.); 4 Department of Pharmacy, “G. d’Annunzio” University of Chieti-Pescara, Via dei Vestini 31, 66100 Chieti, Italy; simone.carradori@unich.it (S.C.); cristina.campestre@unich.it (C.C.); marialucia.gallorini@unich.it (M.G.)

**Keywords:** carbonic anhydrase inhibitors, CORMs, CO release, myoglobin, spectrophotometric assay, molecular hybrids, tendinopathy

## Abstract

Tendinopathy is often described as a complex and multifactorial condition which affects tendons. Tendon disorders are marked by a reduction in mechanical function, accompanied by pain and swelling. At the molecular level, tendinopathy leads to oxidative stress-driven inflammation, increased cell death, disruption of extracellular matrix balance, abnormal growth of capillaries and arteries, and degeneration of collagen formation. Here, we report an innovative approach to modulate oxidative stress during tendinopathy based on sulfonamide-based Carbonic Anhydrase Inhibitors—carbon monoxide releasing molecules (CAI–CORMs) hybrids endowed with dual carbon monoxide (CO) releasing activity and carbonic anhydrase (CA) inhibition. The synthesised compounds have been studied in a model of human Achilles tendon-derived cells stimulated by H_2_O_2_. Among the library, compound **1c** and, to a greater extent, compound **1a**, showed to be extremely effective in terms of restoration of cell metabolic activity and cell proliferation due to their capacity to release CO and inhibit the CA isoforms involved in inflammatory processes in the nanomolar range. Moreover, **1a** can restore collagen type 1 secretion under pro-oxidant conditions.

## 1. Introduction

Tendinopathy describes several diseases characterised by the degeneration of collagen production in the tendon tissue, along with an increase in capillaries and arteries. These illnesses lead to decreased mechanical function, discomfort, and swelling. Uncontrolled inflammation can impede tissue regeneration, causing fibrosis and hampering movement [1]. Numerous in vitro and in vivo studies have shown that the inhibition of oxidative stress-driven inflammation can promote the tenogenic differentiation of tendon stem/progenitor cells, reduce tissue fibrosis and augment tendon repair [2,3]. Due to a dearth of data regarding their effectiveness, there is not a currently recognised therapeutic approach in tendinopathy. Non-steroidal anti-inflammatory drugs (NSAIDs) are widely acknowledged for their ability to relieve acute pain. Nevertheless, there is debate over their application in chronic tendon-related illnesses [4,5]. For these reasons, an innovative approach that can modulate oxidative stress during tendinopathy is urgently needed.

Recent reliable evidence supporting the therapeutic value of low-dose controlled carbon monoxide (CO) delivery through the use of CO-releasing molecules (CORMs) has been found in a large body of research [6,7,8,9,10]. It has now been widely reported that CO acts as a potent anti-inflammatory molecule, both in vitro and in vivo, by selectively activating the hemeoxygenase-1 (HO-1)-related pathway, by inhibiting the expression of the pro-inflammatory cytokines tumour necrosis factor TNF-α and Interleukin (IL)-1β and by increasing production of the anti-inflammatory cytokine IL-10 [11,12,13,14]. Furthermore, it is observed that HO-1 and CO are putatively adaptive in high-altitude human populations and diving mammals that are frequently exposed to hypoxia and/or ischemia-reperfusion events [15,16]. This suggests that endogenous CO and HO provide an evolutionary advantage for hypoxia tolerance and play a crucial role in injury avoidance and cell survival.

The efficiency of oxygen supplying the synovium is poor due to the highly dysregulated synovial microvasculature. This, along with the increased energy demands of activated infiltrating immune cells and inflamed resident cells, leads to a hypoxic microenvironment and favours an increase of reactive oxygen species, boosting oxidative damage, which further promotes inflammation [17]. In a hypoxic microenvironment, abnormal gene expressions lead to tissue injuries [18]. Carbonic anhydrases (CAs) are involved in many pathological conditions underlying hypoxia upon inflammation. Carbonic anhydrase IX has been reported as a potential marker of mechanical stress of joints, ligaments, and tendons during foetal development [19]. In parallel, carbonic anhydrase XII has been reported to play an important role in cartilage, being expressed across all layers of cartilage and functioning as a pH buffer in chondrocytes [20]. Notably, the overexpression of both CA IX and XII in inflamed joints has been reported [21].

In this context, a group of small-molecule hybrids known as CAI–CORM hybrids, which combine a carbonic anhydrase inhibitor (CAI) with a CORM tail section, were described by Berrino et al. [22] as having potential applications in the treatment of rheumatoid arthritis. Furthermore, a series of CAI-CORMs, already characterised and biologically evaluated in a cell model of inflammation established by lipopolysaccharide (LPS) stimulation on mouse macrophages [23], were found to be effective in the counteraction of oxidative stress in human tenocytes [24].

The present work aims to extend the library of sulfonamide-based CAI–CORMs, including internal alkyne systems endowed with different CO-releasing properties, which were also determined using different CO/Mb ratios. The impact of the substituents, the spacer and the presence/absence of the sulfonamide group on the anti-inflammatory and CO-releasing properties of the reported CORMs was also evaluated. Moreover, the effects of the presence of a large excess of a CO acceptor were investigated, aiming at simulating the cellular environment. Finally, this work was designed to offer more insights into the anti-inflammatory properties of CAI–CORMs using a model of H_2_O_2_-stimulated human Achilles tendon-derived cells.

## 2. Results and Discussion

### 2.1. Design and Synthesis

**Figure 1:**
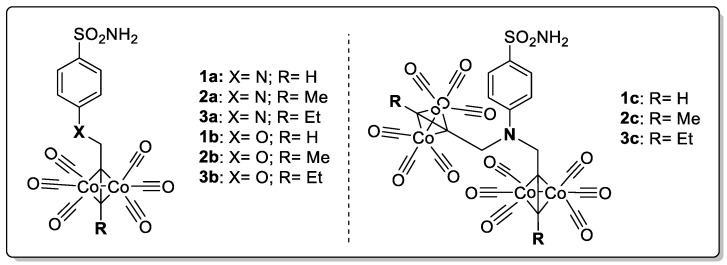
Compounds **1**–**3** (**a**–**c**) reported in this study.

The design of the CAI–CORMs reported in this work was conceived to explore the chemical space around the sulfonamide moiety to assess whether small structural modifications could affect their CA inhibition and CO-releasing profiles and, eventually, their anti-inflammatory properties. Recently, we reported a series of coumarin-based CAI–COMR hybrids as efficient pain-relieving candidates for the treatment of rheumatoid arthritis [25]. In the library, compounds bearing internal alkyne systems were included, and their CO release profiles were compared to the ones from the terminal alkynes. The results showed that this structural modification impacted the CO-releasing properties of the CORMs. Moreover, a stronger pain-relieving activity was observed for “internal” CORMs when compared to the terminal one in the in vivo tests. Thus, in this work, (i) we decided to include terminal and internal alkyne moieties in order to study how this structural modification would impact the biological properties of sulfonamide-based CAI-CORMs; (ii) the bridging atom between the “CORM sphere” and the “drug sphere” was varied; (iii) compounds bearing two CORM moieties were also included. Monosubstituted sulfanilamides (series **a**), disubstituted sulfanilamides (series **c**) and substituted *p*-hydroxy benzenesulfonamides (series **b**) were synthesised by nucleophilic substitution using different alkyl halides (propargyl bromide (series **1**), 1-bromobut-2-yne (series **2**) and 1-bromopent-2-yne (series **3**) (see Supplementary Information File for more details) (Figure 1). The alkyne precursors were then reacted with dicobalt octacarbonyl in tetrahydrofuran (THF) at room temperature (r.t.) according to the standard synthetic protocol to afford the desired dicobalt hexacarbonyl (DCH) CORMs.

**Figure 2:**
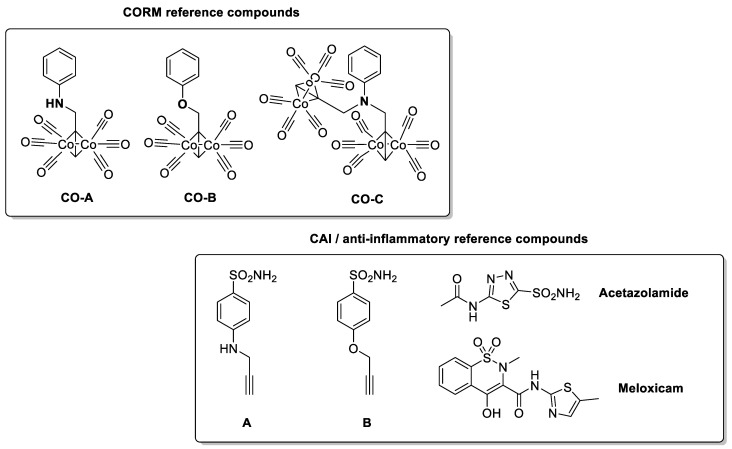
Structures of the reference CORMs compounds **CO-A**, **CO-B**, **CO-C**, the reference CAIs **A**, **B**, **Acetazolamide** and the NSAID **Meloxicam**.

To decipher the contribution of the CO releasing and the CA inhibition in the observed anti-inflammatory activities, compounds bearing only the CORM fraction (**CO-A**, **CO-B** and **CO-C**) and compounds bearing only the CA inhibitory moiety (compounds **A** and **B**) were also synthesised and used as references in the in vitro cellular assays. Acetazolamide and meloxicam were also employed in the tests as reference CA inhibitors and NSAID drugs, respectively (Figure 2).

### 2.2. In Vitro Biological Evaluation

#### 2.2.1. CO Release

**Figure 3:**
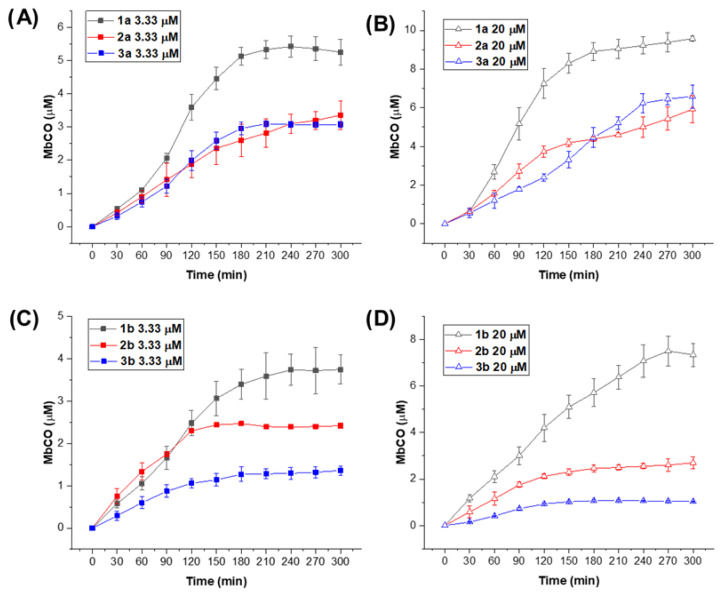
CO-release profiles of compounds **1a**, **2a** and **3a** analysed at 1:6 (**A**) and 1:1 (**B**) CORM-Mb ratios; CO-release profiles of compounds **1b**, **2b** and **3b** analysed at 1:6 (**C**) and 1:1 (**D**) CORM-Mb ratios.

**Figure 4:**
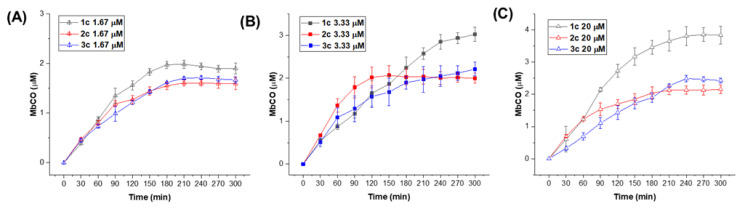
CO-release profiles of compounds **1c**, **2c** and **3c** analysed at 1:12 (**A**), 1:6 (**B**) and 1:1 (**C**) CORM-Mb ratios; (**C**) CO units released by compounds **1c**, **2c** and **3c** after 300 min of incubation working at 1:12 (1.67 μM CORM concentration), 1:6 (3.33 μM CORM concentration) and 1:1 (20 μM CORM concentration) CORM–Mb ratios.

To determine the CO-releasing properties of the designed compounds, the myoglobin carbonylation assay was employed, with some deviations from the standard protocol, as previously reported [26,27,28]. The analysed compounds were incubated with reduced myoglobin (deoxy-Mb), and spectra were recorded every 30 min up to 300 min. The release of CO was assessed by evaluating the formation of carbonylated myoglobin (Mb-CO) over time (Figure 3). Considering that the presence of a large excess of CO acceptor (Mb) would enhance the release of CO from the CORM, different CORM:Mb ratios were explored in the assay (1:1, 1:6 and 1:12, the latter only performed on disubstituted compounds **1c-3c**, Figure 4). The releasing properties of the compounds were also expressed as CO units released after 300 min of incubation with Mb, as reported in Figure S1 (Supplementary Information File).

A first comparison among compounds **1a**–**3a** and **1b**–**3b** in different CORM:Mb conditions (Figure 3) revealed compounds bearing oxygen as a bridging atom (**1b**–**3b**) to be more sensitive to the chemical modifications performed on the CORM section when compared to the nitrogen-containing ones (**1a**–**3a**). In particular, large differences in the MbCO formed over time can be observed within the oxygen-containing series, with the compound bearing the terminal alkyne group (**1b**) being the most efficient, followed by compounds **2b** and **3b**. A similar trend was observed in both 1:1 and 1:6 conditions. As for the nitrogen-containing compounds, compound **1a** showed to be the fastest and most efficient releaser among the series, in agreement with what was observed for the oxygen-containing analogue **1b**, also containing a terminal alkyne group. However, no differences in terms of CO release could be found between the compounds bearing an internal CORM group (**2a** and **3a**), which showed a superimposable profile of MbCO formed over time in both 1:1 and 1:6 experimental conditions. The same results were also observed in the disubstituted series (**1c**–**3c**, Figure 4) in 1:1 CORM:Mb condition.

**Figure 5:**
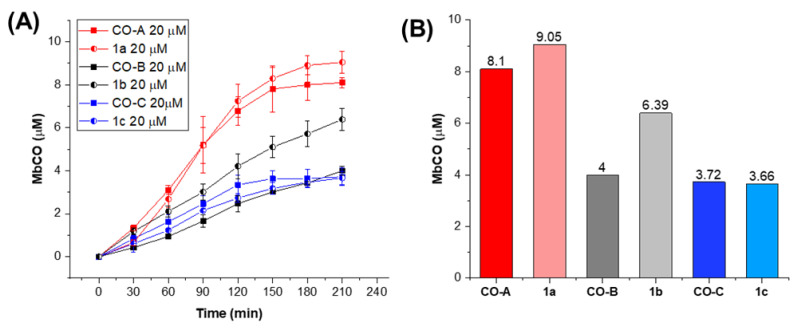
(**A**) CO-release profiles of compounds **1a**, **1b** and **1c** (half-filled circles) and reference compounds **CO-A**, **CO-B** and **CO-C** (filled squares), at 1:1 CORM-Mb ratio; (**B**) MbCO formed (μM) by compounds **1a**, **1b** and **1c** and by the reference compounds **CO-A**, **CO-B** and **CO-C** after 210 min of incubation, at 1:1 CORM–Mb ratio (20 mM CORM concentration).

It is interesting to point out that for the disubstituted compounds, having a higher potential of CO release when compared to the monosubstituted analogues, the presence of a higher amount of Mb acceptor (1:6 and 1:12 conditions) flattened even more the differences among the compounds, although preserving the trend **1c** > **2c** ≥ **3c**. It can be said that less favourable conditions (1:1 CORM:Mb) can be useful to highlight the Structure-Release Relationships (SRRs) from the analysed CORMs, although being not necessarily representative of release observed in the cellular environment, in which a large excess of acceptors is expected (Figure 5).

In order to assess the impact of chemical modifications performed on the drug sphere on the CO release from the analysed CORMs, the analogues of compounds **1a**, **1b** and **1c** devoid of the sulfonamide moiety were also analysed in 1:1 CORM:Mb conditions (compounds **CO-A**, **CO-B** and **CO-C**, respectively). Interestingly, the insertion of the sulfonamide group on the aryl moiety of the CORM was shown to significantly enhance the CO release only for compound **1b**, bearing an oxygen bridge atom, whereas the CO release from nitrogen-containing analogues was not impacted by this chemical modification (with only a slight enhancement recorded from compound **1a** when compared to **CO-A**). Once again, these results confirmed the assumption that compounds bearing oxygen as bridge atoms are more sensitive to the surrounding chemical environment when compared to nitrogen-containing ones. Interestingly, this observation was recently highlighted by some of us in a joined theoretical and experimental study aimed at understanding more in-depth the CO-releasing mechanism from the DCH CORM family, which is still largely debated nowadays [29].

Overall, analysis of the CO units released after 300 min of incubation with Mb (See Figure S1, Supporting Information) showed that compounds **1a** and, to a greater extent, compound **1c** are the best releasers among the series, being able to release 1.6 and 2.3 CO units when analysed in 1:6 and 1:12 CORM:Mb ratios, respectively.

For the sake of clarity, we want to stress that the conditions used to perform the myoglobin assay are clearly different from the physio/pathological ones in tenocyte cultures, especially under pro-oxidative stress. However, the myoglobin assay remains a gold standard in determining the CO release from CORMs, which is essential in comparing CORM profiles and gathering some preliminary information about their relative effectiveness in terms of CO release in cells.

#### 2.2.2. CA Inhibition

The inhibitory properties of the CAI−CORM dual hybrids **1**–**3** (**a**–**c**) were appraised against the CAs I, II, IX, and XII isoforms by employing the stopped-flow CO_2_ hydrase assay [30,31]. Although CA I and II are usually considered off-targets for the treatment of inflammatory-based diseases based on CA inhibitors, CA IX (and marginally CA XII) coupled with the intracellular hCA II, thus participating in a complex pH regulation machinery based on the metabolic switch typical of hypoxic cells [32]. Therefore, inhibition of CA II is also desired. Concerning CA IX and CA XII, recently reported works demonstrated their contribution to inflammatory diseases, thus corroborating their use as targets for the treatment of these diseases [21,23]. In light of the above, we chose these isoforms to be tested towards our library. The inhibitory activity data, compared to those of the standard sulfonamide inhibitor acetazolamide (**AAZ**), are reported in Table 1, while the inhibition data of the alkyne precursors are reported in the Supplementary Information File (Table S1). By comparing the *K*_I_ data of the CAI−CORM with the alkyne precursors, it is possible to observe that the insertion of the CO-releasing moiety affected the inhibitory activity towards the tested isoforms (Table 1 and Table S1). Overall, the CAI–CORM resulted in being less effective than the parent alkynes towards CA I, with the *K*_I_ values falling in the sub-micromolar/micromolar range (Table 1). Similar results were observed also for CA II, albeit some CAI-CORM maintained a nanomolar inhibition of this isoform. In particular, compound **2b** was the most effective CA II inhibitor of the entire library (**2b**, *K*_I_ CA II = 7.0 nM), followed by **2c** and **3c**, which shared the same *K*_I_ value of 9.5 nM. With regards to the inhibition data of the molecules provided with nitrogen bridge atoms (**a1**–**3** and **c1**–**3**), it is possible to note that the disubstituted sulfanilamides exhibited a better performance towards CA II than the monosubstituted ones. As a matter of fact, the insertion of a second substituent bound to the nitrogen led to a 247- and 446-fold increase of inhibitory activity for the couples **2a**/**2c** and **3a**/**3c**, respectively (Table 1). This trend was less pronounced for the couple **1a**/**1c**, thus highlighting also the importance of the substituent group. Indeed, the increasing of the alkyne moiety’s length, i.e., moving from terminal (**1a**/**1c**, R = H) to internal alkyne (**2**–**3a**, R = Me; **2**–**3c**, R = Et), had a detrimental effect for monosubstituted compounds (**a1**–**3**), while improved the inhibitory activity for the disubstituted ones (**c1**–**3**). Considering the compounds bearing oxygen as bridge moiety (**b1**–**3**), we already reported the derivative **2b** as the most effective against hCA II. The elongation of the alkyl chain bound to the alkyne moiety led to the poor inhibitor **3b** (**3b**, *K*_I_ CA II = 600.1 nM), while the derivative **1b** endowed with terminal alkyne displayed nanomolar affinity against hCA II (**1b**, *K*_I_ CA II = 40.0 nM). Overall, the inhibition of hCA IX (and XII) resulted in an improvement in the presence of the CO-releasing moiety with respect to the alkyne precursors (see Table 1 and Table S1). Indeed, apart from compound **1a**, all the CAI-CORMs inhibited hCA IX in the nanomolar range with potency following the trend **1b-c** > **2b-c** > **3b-c** (Table 1). As a matter of fact, compounds **1b** (*K*_I_ CA IX = 5.4 nM) and **1c** (*K*_I_ CA IX = 6.2 nM) bearing the terminal alkyne were the most effective among the substituted *p*-hydroxy derivatives and disubstituted sulfanilamides, respectively.

Compounds belonging to the monosubstituted sulfanilamides (**a1**–**3**) did not abide by this general trend, **1a** being ineffective against hCA IX, while derivatives **2a** and **3a** exhibited comparable *K*_I_ of 27.8 nM and 26.5 nM, respectively. Similar results were observed for CA XII isoform (Table 1). Indeed, while compound **1a** was ineffective against CA XII (**1a**, *K*_I_ CA XII = 10,000 nM), compounds **2a** and **3a** potently inhibited this isoform with *K*_I_ values in the low nanomolar range (**2a**, *K*_I_ CA XII = 4.6 nM; **3a**, *K*_I_ = CA XII 2.1 nM). Compound **1b,** the most potent CA IX inhibitor, also exhibited the best performance against CA XII, the inhibitory activity falling in the sub-nanomolar range (**1b**, *K*_I_ CA XII = 0.9 nM). Passing from the terminal alkyne (**1b**, R = H, Table 1) to the internal one of compound **2b** (R = CH_3_, Table 1) led to the impairment of the inhibitory activity; however, further elongation of the alkyl group in compound **3c** (R = Et), restored the low nanomolar affinity (**3c**, *K*_I_ CA XII = 3.8 nM). Concerning the disubstituted sulfanilamides (**c1**–**3**), their CA XII inhibition followed the trend **1c** > **2c** > **3c**, previously observed for CA IX. Indeed, while compound **1c** exhibited low nanomolar activity against this isoform (**1c**, *K*_I_ CA XII = 3.8 nM), the analogues provided with internal alkyne (**2c** and **3c**) showed slightly impaired activity, albeit still falling in the nanomolar range.

#### 2.2.3. Cellular Assays on Human Achilles Tendon-Derived Cells

The derivatives **1**–**3** (**a**–**c**) and the reference compounds **CO-A**, **CO-B-I**, **CO-C**, **A** and **B** were appraised on human tenocytes to investigate their antioxidant properties and extracellular matrix (ECM) remodelling potential in terms of enhancement of cell metabolic activity, under basal and pro-oxidant conditions, modulation of cell cycle and collagen type 1 release.

**Figure 6:**
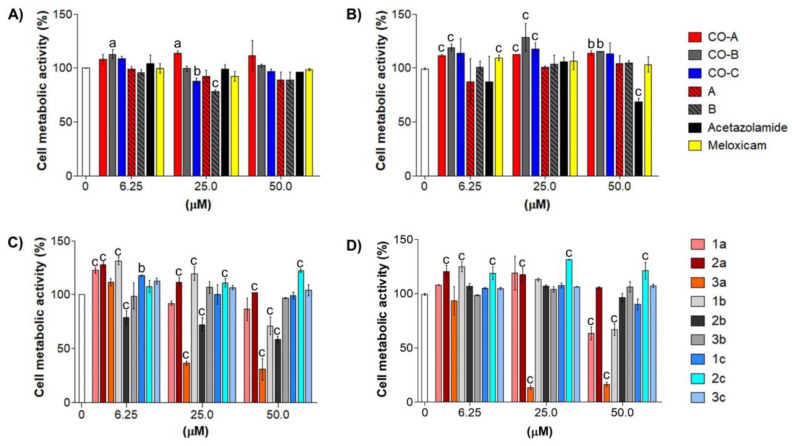
Cell metabolic activity of tenocytes in the presence of reference compounds **CO-A**, **CO-B**, **CO-C**, acetazolamide and meloxicam and compounds **1**–**3** (**a**–**c**) after 24 h of exposure (6.25–50 µM). (**A**,**C**) Bar graphs represent samples without an H_2_O_2_ pre-incubation. (**B**,**D**) Bar graphs show samples pre-incubated with H_2_O_2_ (100 µM for 3 h) and afterwards treated with compounds for 24 h. Untreated cultures (0 µM) are set as 100%. Each experiment was performed three times in triplicates per experimental condition (n = 9). a = *p* < 0.01; b = *p* < 0.001; c = *p* < 0.0001 between treated samples and the untreated one.

Cell migration and proliferation are key events involved in many biological processes, including embryological development, tissue formation, and healing [34]. Since an augmented metabolism under basal conditions is tightly related to cell proliferation and functions, compounds were first administered under basal conditions to investigate biocompatibility and to assess whether they are able to enhance the cell metabolic activity of tenocytes [35]. Reference compounds are only weakly effective in terms of cell metabolic activity after 24 h, except for **CO-C** and compound **B** at 25 µM (Figure 6A). On the other hand, **1a**, **2a**, **1b** and **2c** significantly increase cell metabolic activity in the same experimental conditions. In parallel, **3a** and **2b** reveal cytotoxicity (Figure 6C).

**Figure 7:**
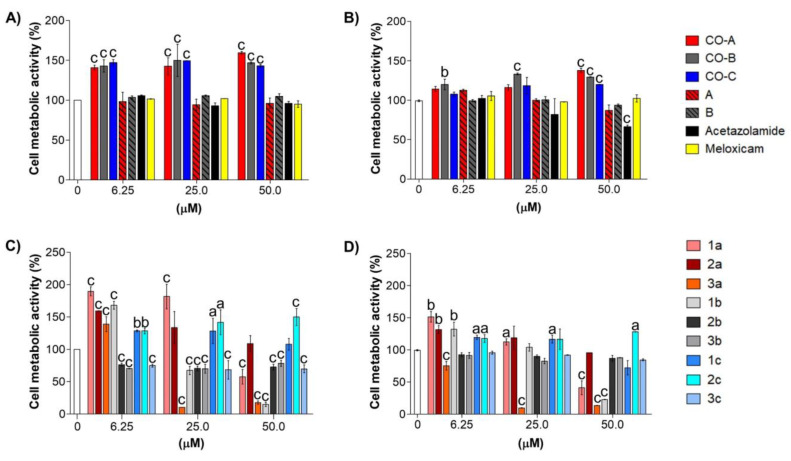
Cell metabolic activity of tenocytes in the presence of reference compounds **CO-A**, **CO-B-I**, **CO-C**, acetazolamide and meloxicam and compounds **1**–**3** (**a**–**c**) after 48 h of exposure (6.25–50 µM). (**A**,**C**) Bar graphs represent samples without an H_2_O_2_ pre-incubation. (**B**,**D**) Bar graphs show samples pre-incubated with H_2_O_2_ (100 µM for 3 h) and afterwards treated with compounds for 48 h. Untreated cultures (0 µM) are set as 100%. Each experiment was performed three times in triplicates per experimental condition (n = 9). a = *p* < 0.01; b = *p* < 0.001; c = *p* < 0.0001 between treated samples and the untreated one.

After 48 h of exposure to basal conditions, **CO-A**, **CO-B** and **CO-C** are extremely active in terms of cell metabolic activity increase, whereas **A**, **B**, acetazolamide, and meloxicam are still ineffective (Figure 7A). As for test compounds (Figure 7C), **1a** well increases cell metabolic activity at the lower concentrations, being cytotoxic at 50 µM. Compound **2a** increases tenocyte metabolic activity to a lesser extent than **1a**, also revealing a dose-dependent decrease in the percentages of active cells over the concentration range but remaining biocompatible. Compound **1b** displays cytotoxicity from 25 µM, whereas **2b**, **3b** and **3c** are cytotoxic, already at the lowest concentration, where percentages of cell metabolic activity significantly decreased compared to the untreated control. In parallel, **1c** and **2c** highlight the best results among the series, being constantly effective on cell metabolic activity over the concentrations.

Sustained oxidative stress and the subsequent activation of redox-sensitive molecular pathways have been reported as the major factors responsible for tendon inflammation and failure of tendon healing [2,36]. In a pro-oxidant environment and after 24 h of exposure to reference compounds (Figure 7B), only **CO-A**, **CO-B**, **CO-C,** and meloxicam are capable of increasing cell metabolic activity. It is worth noting that acetazolamide displays cytotoxicity at the highest dose. In parallel, the best test compounds able to increase cell metabolic activity after 24 h (Figure 6D) over the concentration range are **2a** and **2c**. Compound **1a** and, to a greater extent, compound **3a** are cytotoxic at the highest concentrations used. After 48 h, only **CO-B** can increase cell metabolic activity over the concentration range (Figure 7B). **CO-A** and **CO-C** increase cell metabolic activity only at 50 µM. Again, acetazolamide is registered cytotoxic at the highest concentration. As for test compounds, **2c** is the best compound in terms of cell metabolic activity increase because the metabolic rate is maintained high over the concentration range, whereas other test compounds (i.e., **1a**), although initially promising, reveal cytotoxicity at the highest concentrations (Figure 7D).

Due to cell metabolic activity resulting in the lower concentration range and their CO-release profile and CA inhibition data, compounds **1a** and **1c** were chosen for further experiments regarding the analysis of the cell cycle progression and the release of collagen type 1. Compound **A** and acetazolamide were administered as reference compounds. All the compounds were used at the concentration of 6.25 µM for 48 h under pro-oxidant conditions (pre-incubation with H_2_O_2_).

**Figure 8:**
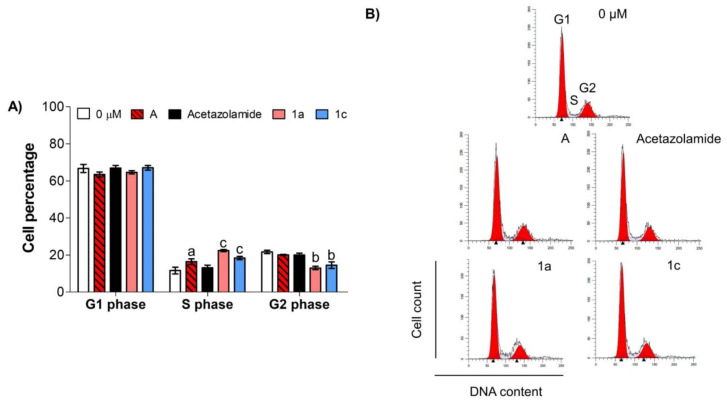
Cell cycle analysis in the presence of reference compounds **A** and acetazolamide and compounds **1a** and **1c** after 48 h of exposure at 6.25 µM. (**A**) The bar graph shows cell percentages in the various phases of the cell cycle (G1, S, and G2) of tenocytes pre-incubated with H_2_O_2_ 100 μM for 3 h and afterwards exposed to compounds for 48 h. Each experiment was performed two times in triplicates per experimental condition (n = 6). a = *p* < 0.01; b = *p* < 0.001; c = *p* < 0.0001 between treated samples and the untreated one (0 µM). (**B**) Cell cycle profiles represented by fluorescence emission peaks obtained after the propidium iodide staining (*y*-axis = cell count; *x*-axis = propidium iodide fluorescence emission in the FL3 channel-DNA content).

Tenocytes only pre-incubated with H_2_O_2_ and afterwards exposed to a growth medium display a typical cell cycle profile ascribable to cells in an acute pro-oxidant condition, with their G2 phase weakly increased. Compounds **1a** and **1c** weakly but significantly counteract the H_2_O_2_-induced G2 increase, whereas **A** and acetazolamide are ineffective (Figure 8A). Increasing cell metabolism and proliferation are particularly important for tendon tissue repair after the acute inflammatory phase [37]. As a matter of fact, tendon healing occurs in different stages, among which the consolidation phase involves both high cell metabolism and proliferation. After that, in the remodelling phase, cells decrease their proliferation rate and start to produce collagen type I [24]. Being **1a** the most effective compound in terms of modulation of cell cycle progression, the amount of secreted collagen type I was measured in the presence of this compound.

**Figure 9:**
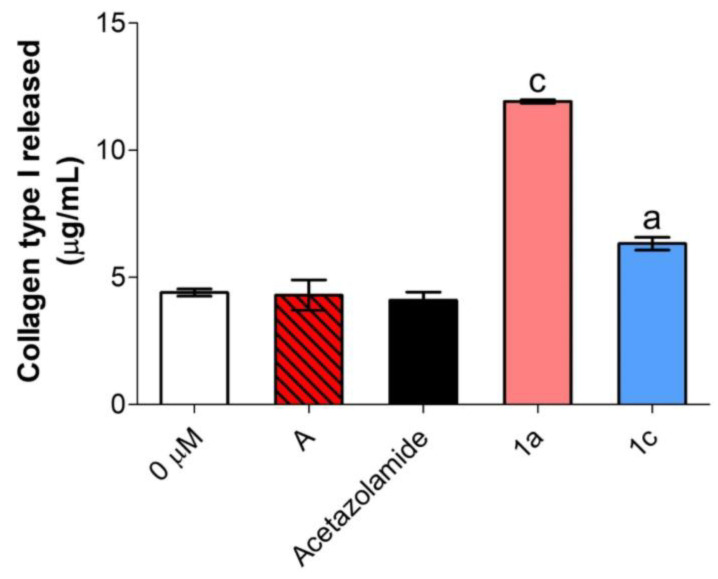
Collagen type I secretion from tenocytes in the presence of compound **A**, acetazolamide and compounds **1a** and **1c** after 48 h of exposure at 6.25 µM. Bar graphs show collagen type I concentration obtained by ELISA assay in cell supernatants (μg/mL) of tenocytes pre-incubated with H_2_O_2_ 100 μM for 3 h and afterwards exposed to compounds. Values are normalised on MTT data from the same experiment. Each ELISA test was performed three times in duplicates per experimental condition (n = 6). a = *p* < 0.01; b = *p* < 0.001; c = *p* < 0.0001 between treated samples and the untreated one (0 µM).

Healing is a complex process, including three overlapping stages: inflammation, proliferation, and remodelling. During the proliferative and remodelling phases of healing, tenocytes proliferate and begin to produce, secrete, and crosslink fibrillar collagens [38]. Collagen type I is the major collagen isoform secreted by tenocytes, and higher collagen type I production is reported to be a key reason for improved tendon healing [39,40]. In our experimental conditions, **1a** dramatically increases its secretion almost three times compared to untreated cells. Likewise, compound **1c** weakly but significantly increases collagen type 1 secretion (Figure 9). Compound **A** and acetazolamide do not enhance the secretion when compared to the untreated samples.

## 3. Conclusions

Here, we report a novel, innovative approach to modulate oxidative stress during tendinopathy, based on the administration in vitro of sulfonamide-based CAI–CORM hybrids endowed with both CO-releasing activity and carbonic anhydrase inhibition in H_2_O_2_-stimulated Achilles tendon-derived cells.

Among the appraised compounds, derivatives **1a** (to a greater extent) and **1c** exhibited the best performance as antioxidant agents. The CA inhibition profiles of compounds **1a** and **1c** against the cytosolic isoforms CA I and CA II are quite similar (*K*_I_ values in the medium/high nanomolar range), whereas large differences can be found against the transmembrane isoforms hCA IX and XII. In terms of CO release, **1a** and **1c** showed a similar profile in a large excess of acceptor, which more closely resembles the cellular environment. However, **1c** is slightly (1.4-fold) more efficient in terms of CO units released than **1a** when analysed in comparable conditions (1:12 and 1:6, respectively). Therefore, the biological activities observed for compounds **1a** and **1c** are most likely to be ascribed to their CO-releasing properties rather than to their CA inhibitory activities.

Interestingly, this study confirms in vitro the promising results that we previously obtained in the in vivo studies conducted on compound **1a** in a model of rheumatoid arthritis [22,33], allowing us to gather more insight into its anti-oxidative stress-driven molecular mechanisms in the musculoskeletal compartment.

## 4. Experimental Protocols

### 4.1. General

Anhydrous solvents and all reagents were purchased from Sigma-Aldrich (Milan, Italy), Alfa Aesar (Milan, Italy) and TCI (Milan, Italy). All reactions involving air- or moisture-sensitive compounds were performed under a nitrogen atmosphere using dried glassware and syringes techniques to transfer solutions. Nuclear magnetic resonance spectra (^1^H-NMR: 400 MHz; ^13^C-NMR: 100 MHz) were recorded in DMSO-*d*_6_ using an Avance III 400 MHz spectrometer (Bruker, Milan, Italy). Chemical shifts are reported in parts per million (ppm), and the coupling constants (J) are expressed in Hertz (Hz). Splitting patterns are designated as follows: s, singlet; d, doublet; t, triplet; q, quadruplet; m, multiplet; brs, broad singlet; dd, double of doublets. The assignment of exchangeable protons (SO_2_NH_2_) was confirmed by the addition of D_2_O. Analytical thin-layer chromatography (TLC) was carried out on silica gel F-254 plates (Merck, Milan, Italy). The solvents used in MS measures were acetone, acetonitrile (Chromasolv grade), purchased from Sigma–Aldrich and mQ water 18 MΩ cm, obtained from Millipore’s Simplicity system (Milan, Italy).

### 4.2. Chemistry

#### 4.2.1. General Procedure for the Preparation of Final Compounds **1**–**3** (**a**–**c**) and the Reference Compounds **CO-(A-C)**

**Scheme 1:**
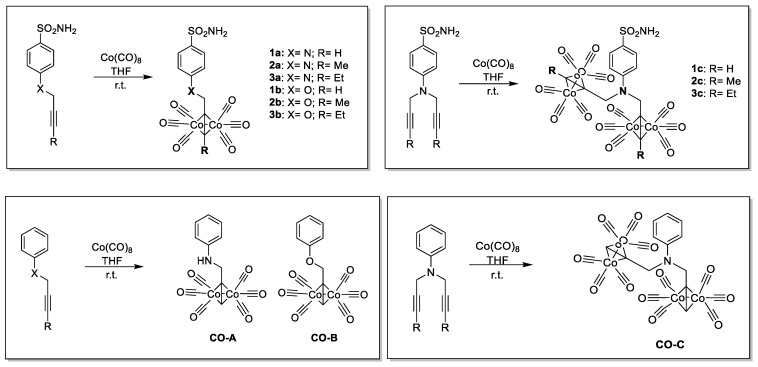
Synthesis of the compounds **1**–**3** (**a**–**c**) and CO-A, CO-B and CO-C.

The appropriate alkynyl derivatives (0.1 g, 1.0 eq.) were dissolved in THF (5 mL), and then dicobalt octacarbonyl (1.05 eq. or 2.1 eq. depending on the alkyne considered) was added. The black mixture was stirred at r.t. for 40 min. (TLC monitoring). Then, SiO_2_ was added, and the solvent was removed under vacuum to give a black, solid residue, which was purified by silica gel column chromatography, eluting with the appropriate mixture of EtOAc in *n*-Hexane to afford the desired compounds (Scheme 1).

#### 4.2.2. Characterisation Data of Final Compounds **1**–**3** (**a**–**c**) and the Reference Compounds **CO-(A-C)**

*4-(prop-2-ynylamino)benzenesulfonamide hexacarbonyldicobalt* (**1a**)

The titled compound **1a** was obtained according to the general procedure, using 4-(prop-2-ynylamino)benzenesulfonamide **A** as the starting material. Purified eluting with EtOAc/n-Hex 40% *v*/*v*. 73% yield; silica gel TLC R*f* = 0.54 (EtOAc/n-Hex 60% *v*/*v*); δ_H_ (400 MHz, DMSO-*d*_6_): 4.56 (2H, d, *J* = 6.4, CH_2_), 6.66 (3H, m, 1 × C*H*, 2 × Ar-*H*), 6.96 (2H, br.s, exchange with D_2_O, SO_2_NH_2_), 7.09 (1H, t, *J* = 6.5, exchange with D_2_O, N*H*), 7.53 (2H, d, *J* = 8.3, 2 × Ar-*H*). Experimental in agreement with reported data [22].

*4-(but-2-yn-1-ylamino)benzenesulfonamide hexacarbonyldicobalt* (**2a**)

The titled compound **2a** was obtained according to the general procedure, using 4-(but-2-yn-1-ylamino)benzenesulfonamide **A-II** as the starting material. Purified eluting with EtOAc/n-Hex 30% *v*/*v*. 87% yield; silica gel TLC R*f* = 0.20 (EtOAc/n-Hex 30% *v*/*v*); δ_H_ (400 MHz, DMSO-*d*_6_): 2.54 (3H, s, overlapped with DMSO signal, CH_3_), 4.58 (2H, d, J = 6.5, CH_2_), 6.72 (2H, d, J = 8.4, Ar-H), 6.95 (2H, br.s, exchange with D_2_O, SO_2_NH_2_), 7.11 (1H, t, J = 6.7, exchange with D_2_O, NH), 7.54 (2H, d, J = 8.3, Ar-H); δ_C_ (100 MHz, DMSO-*d*_6_): 20.4, 44.3, 93.7, 97.7, 111.1, 127.2, 130.8, 150.4, 199.8.

*4-(pent-2-yn-1-ylamino)benzenesulfonamide hexacarbonyldicobalt* (**3a**)

The titled compound **3a** was obtained according to the general procedure, using 4-(pent-2-yn-1-ylamino)benzenesulfonamide **A-III** as the starting material. Purified eluting with EtOAc/n-Hex 30% *v*/*v*. 78% yield; silica gel TLC R*f* =0.42 (EtOAc/n-Hex 50% *v*/*v*); δ_H_ (400 MHz, DMSO-*d*_6_): 1.16 (3H, t, J = 7.3, CH_3_), 2.74 (2H, q, J = 7.4, CH_2_), 4.61 (2H, d, J = 6.4, CH_2_), 6.71 (2H, d, J = 8.3, Ar-H), 6.96 (2H, br.s, exchange with D_2_O, SO_2_NH_2_), 7.11 (1H, t, J = 6.7, exchange with D_2_O, NH), 7.52 (2H, d, J = 8.3, Ar-H); δ_C_ (100 MHz, DMSO-*d_6_*): 15.5, 26.1, 44.4, 97.1, 101.9, 111.0, 127.2, 130.8, 150.4, 200.0.

*4-(Prop-2′-ynyloxy)benzenesulfonamide hexacarbonyldicobalt* (**1b**)

The titled compound **1b** was obtained according to the general procedure previously reported using 4-(prop-2′-ynyloxy)benzenesulfonamide **B** as the starting material. Purified eluting with EtOAc/n-Hex 30% *v*/*v*. 82% yield; silica gel TLC R*f* = 0.43 (EtOAc/n-Hex 40% *v*/*v*); δ_H_ (400 MHz, DMSO-*d*_6_) 5.40 (2H, s, CH_2_), 6.83 (1H, s, CH), 7.13 (2H, d, J = 8.8, Ar-H), 7.24 (2H, br.s, exchange with D_2_O, SO_2_NH_2_), 7.77 (2H, d, J = 8.8, Ar-H). Experimental in agreement with reported data [22].

*4-(But-2-yn-1-yloxy)benzenesulfonamide hexacarbonyldicobalt* (**2b**)

The titled compound **2b** was obtained according to the general procedure, using 4-(but-2-yn-1-yloxy)benzenesulfonamide **B-II** as the starting material. Purified eluting with EtOAc/n-Hex 30% *v*/*v*. 54% yield; silica gel TLC R*f* = 0.15 (EtOAc/n-Hex 30% *v*/*v*); δ_H_ (400 MHz, DMSO-*d*_6_): 2.64 (3H, s, CH_3_), 5.43 (2H, s, CH_2_), 7.14 (2H, d, J = 8.4, Ar-H), 7.22 (2H, br.s, exchange with D_2_O, SO_2_NH_2_), 7.78 (2H, d, J = 8.3, Ar-H); δ_C_ (100 MHz, DMSO-*d*_6_): 20.0, 68.0, 91.1, 99.4, 114.5, 127.6, 136.6, 160.2, 199.6.

*4-(Pent-2-yn-1-yloxy)benzenesulfonamide hexacarbonyldicobalt* (**3b**)

The titled compound **3b** was obtained according to the general procedure, using 4-(but-2-yn-1-yloxy)benzenesulfonamide **B-III** as the starting material. Purified eluting with EtOAc/n-Hex 30% *v*/*v*. 45% yield; silica gel TLC R*f* = 0.20 (EtOAc/n-Hex 30% *v*/*v*); δ_H_ (400 MHz, DMSO-*d*_6_): 1.25 (3H, t, *J* = 7.3, CH_3_), 2.86 (2H, q, J = 7.3, CH_2_), 5.43 (2H, s, CH_2_), 7.14 (2H, d, J = 8.5, Ar-H), 7.22 (2H, br.s, exchange with D_2_O, SO_2_NH_2_), 7.78 (2H, d, J = 8.5, Ar-H); δ_C_ (100 MHz, DMSO-*d*_6_):15.5, 26.2, 68.3, 91.2, 99.4, 114.5, 127.7, 136.6, 160.3, 199.7.

*4-(Diprop-2′-ynylamino)benzenesulfonamide hexacarbonyldicobalt* (**1c**)

The titled compound **1c** was obtained according to the general procedure previously reported using 4-(diprop-2′-ynylamino)benzenesulfonamide **C** as the starting material. Purified eluting with EtOAc/n-Hex 20% *v*/*v*. 79% yield; silica gel TLC R*f* = 0.43 (EtOAc/n-Hex 30% *v*/*v*); δ_H_ (400 MHz, DMSO-*d*_6_) 4.91 (4H, br s, 2 × CH_2_), 6.93 (6H, m, 2 × CH, 2 × Ar-H, exchange with D_2_O, SO_2_NH_2_), 7.64 (2H, d, J = 8.8, Ar-H). Experimental in agreement with reported data [22].

*4-(Di(but-2-yn-1-yl)amino)benzenesulfonamide hexacarbonyldicobalt* (**2c**)

The titled compound **2c** was obtained according to the general procedure, using 4-(di(but-2-yn-1-yl)amino)benzenesulfonamide **C-II** as the starting material. Purified eluting with EtOAc/n-Hex 20% *v*/*v*. 52% yield; silica gel TLC R*f* = 0.46 (EtOAc/n-Hex 30% *v*/*v*); δ_H_ (400 MHz, DMSO-*d*_6_): 2.62 (6H, s, 2 × CH_3_), 4.93 (4H, s, 2 × CH_2_), 7.00 (2H, d, J = 8.6, Ar-H), 7.08 (2H, br.s, exchange with D_2_O, SO_2_NH_2_), 7.65 (2H, d, J = 8.4, Ar-H); δ_C_ (100 MHz, DMSO-*d*_6_): 20.1, 50.6, 91.4, 94.4, 111.4, 126.2, 131.7, 147.9, 200.1.

*4-(Di(pent-2-yn-1-yl)amino)benzenesulfonamide hexacarbonyldicobalt* (**3c**)

The titled compound **3c** was obtained according to the general procedure, using 4-(di(pent-2-yn-1-yl)amino)benzenesulfonamide **C-III** as the starting material. Purified eluting with EtOAc/n-Hex 20% *v*/*v*. 65% yield; silica gel TLC R*f* = 0.50 (EtOAc/n-Hex 30% *v*/*v*); δ_H_ (400 MHz, DMSO-*d*_6_): 1.18 (6H, t, *J* = 7.7, 2 × CH_3_), 2.80 (4H, q, J = 7.5, 2 × CH_2_), 4.97 (4H, s, 2 × CH_2_), 7.00 (2H, d, J = 8.5, Ar-H), 7.06 (2H, br.s, exchange with D_2_O, SO_2_NH_2_), 7.64 (2H, d, J = 8.7, Ar-H); δ_C_ (100 MHz, DMSO-*d*_6_): 15.5, 26.1, 51.6, 91.9, 102.7, 111.4, 127.1, 128.2, 148.1, 199.7.

*N-(prop-2-yn-1-yl)aniline hexacarbonyldicobalt* (**CO-A**)

The titled compound **CO-A** was obtained according to the general procedure, using N-(prop-2-yn-1-yl)aniline as the starting material. Purified eluting with EtOAc/n-Hex 5% *v*/*v*. 56% yield; silica gel TLC R*f* = 0.43 (EtOAc/n-Hex 20% *v*/*v*); δ_H_ (400 MHz, DMSO-*d*_6_): 4.50 (2H, d, *J* = 6.5, CH_2_), 6.32 (1H, t, J = 6.6, CH), 6.59 (4H, m, 3 × Ar-H, 1 × NH, exchange with D_2_O), 7.08 (2H, d, J = 7.8, Ar-H); δ_C_ (100 MHz, DMSO-*d*_6_): 44.9, 74.1, 95.2, 112.3, 116.1, 128.8, 147.3, 199.9.

*(Prop-2-yn-1-yloxy)benzene hexacarbonyldicobalt* (**CO-B**)

The titled compound **CO-B** was obtained according to the general procedure, using (prop-2-yn-1-yloxy)benzene as the starting material. Purified eluting with EtOAc/n-Hex 2% *v*/*v*. 56% yield; silica gel TLC R*f* = 0.65 (EtOAc/n-Hex 3% *v*/*v*); δ_H_ (400 MHz, DMSO-*d*_6_): 5.29 (2H, s, CH_2_), 6.79 (1H, s, CH), 7.01 (3H, m, Ar-H), 7.32 (2H, t, J = 8.0, Ar-H); Experimental in agreement with reported data [41].

*N,N-di(prop-2-yn-1-yl)aniline hexacarbonyldicobalt* (**CO-C**)

The titled compound **CO-C** was obtained according to the general procedure, using (N,N-di(prop-2-yn-1-yl)aniline as the starting material. Purified eluting with EtOAc/n-Hex 1% *v*/*v*. 45% yield; silica gel TLC R*f* = 0.33 (EtOAc/n-Hex 1% *v*/*v*); δ_H_ (400 MHz, DMSO-*d*_6_): 4.83 (4H, s, 2 × CH_2_), 6.69 (1H, t, J = 7.4, Ar-H), 6.82 (4H, m, 2 × CH, 2 × Ar-H), 7.21 (2H, t, J = 7.8, Ar-H).

### 4.3. CA Inhibition

An Applied Photophysics stopped-flow instrument was used for assaying the CA-catalysed CO_2_ hydration activity [31,42] Phenol red (at a concentration of 0.2 mM) was used as indicator, working at the absorbance maximum of 557 nm, with 10 mM Hepes (pH 7.5) as buffer, 0.1 M Na_2_SO_4_ (to keep constant ionic strength), following the CA-catalysed CO_2_ hydration reaction for a period of 10–100 s. The CO_2_ concentrations ranged from 1.7 to 17 mM for the determination of the kinetic parameters and inhibition constants. For each inhibitor, at least six traces of the initial 5–10% reaction were used to determine the initial velocity. The uncatalysed rates were determined in the same manner and subtracted from the total observed rates. Stock solutions of inhibitors (10 mM) were prepared in distilled-deionised water with 10% DMSO, and dilutions up to 0.001 mM were performed thereafter with the assay buffer. The inhibitor and enzyme solutions were preincubated together for 15 min (standard assay at room temperature) prior to assay to allow for the formation of the E–I complex. The inhibition constants were obtained by non-linear least-squares methods using PRISM 3 and the Cheng–Prusoff equation and represent the mean from at least three different determinations. Enzyme concentrations in the assay system were in the range of 5–12 nM.

### 4.4. CO-Release Assay

All the employed reagents were of analytical grade and acquired from Merck (Milan, Italy). Gaseous CO was obtained from Rivoira (Milan, Italy). A Shimadzu UV1900 UV-Vis Spectrophotometer from 275 to 700 nm at the scanning rate of 200 nm/min was used to record UV-Vis absorption spectra in a disposable plastic cuvette (path length 0.44 cm). The second derivative spectra were generated with the Origin Lab software, and the Savitzky–Golay method was applied using 25 data points for the differentiation process. Neither an increase nor a decrease in the number of points caused changes in the wavelength or in the bandwidth. Lyophilised horse heart Mb was dissolved in phosphate-buffered saline flushed with N_2_ (PBS, 0.01 M, pH 7.4 to a 20–22 μM final concentration). Two millilitres of this freshly prepared stock solution were placed in a cuvette to record the UV-Vis absorption spectrum of met-Mb. Next, the solutions were divided into two halves: 10 μL of sodium dithionite (30 mg/mL) was added to the first half (reference), and the UV–Vis spectrum of deoxy-Mb was registered. After that, the solution was flushed with CO gas, and the Mb-CO spectrum was acquired. Sodium dithionite was added to the second half (sample), and a spectrum was recorded. Afterwards, a CORM DMSO solution was added to a final CORM concentration of 1.67 μM, 3.33 μM or 20 μM and gently mixed. The solution was covered with 300 μL of light mineral oil to avoid CO escaping and oxygenation of Mb, and the absorption spectrum was recorded at t = 0. Spectra were acquired every 30 min for 300 min, keeping the sample at 37 °C. When necessary, a freshly prepared sodium dithionite solution was added. After 300 min, the total Mb concentration at the end of the assay was determined by flushing the sample with CO gas. Mb-CO concentration at each time point was determined, as previously reported [16]. Each experiment was replicated three times, and the data were expressed as mean ± SEM.

### 4.5. Cellular Assays

#### 4.5.1. Cell Culture

Human Achilles tendon-derived tenocytes (#TEN-F) were purchased by ZenBio Inc. (Durham, NC, USA) and maintained in complete alpha-MEM (EuroClone, Milan, Italy) supplemented with 10% of heat-inactivated FBS (Gibco, Thermo Fisher Scientific, Waltham, MA, USA) and 1% penicillin/streptomycin (EuroClone, Milan, Italy) at 37 °C and 5% CO_2_ and used from passage 3 up to passage 6.

#### 4.5.2. Cell Exposure to Compounds

Cells were seeded in 96-well plates (0.5 × 10^4^/well) or 6-well plates (0.5 × 10^5^/well) (ThermoFisher Scientific, Waltham, MA, USA) and left to adhere overnight at 37 ˚C and 5% CO_2_. For cell metabolic activity experiments (MTT test), tenocytes were treated with increasing concentrations of compounds (range 0–50 µM) for 24 and 48 h. Compounds were dissolved in DMSO to obtain a 200 mM stock solution, and they were afterwards diluted in complete alpha-MEM (DMSO final concentration = 0.1%) for further analyses. In a second set of MTT experiments, tenocytes were pre-incubated with 100 µM H_2_O_2_ for 3 h. After that, the pre-incubation medium was discarded and replaced with a fresh one containing the proper compound at increasing concentrations for 24 and 48 h. At the established time points, samples were processed for further analyses.

#### 4.5.3. Cell Metabolic Activity (MTT Test)

Tenocytes were seeded in 96 well tissue culture-treated plates (Falcon^®^, Corning Incorporated, Brooklyn, NY, USA) at 0.5 × 10^4^ cells/well. Untreated cells (0 µM) were set as control (100% of cell metabolic activity). At the established time points, the exposure media were replaced by fresh medium containing 3-(4,5-dimethylthiazol-2-yl)-2,5-diphenyltetrazolium bromide (MTT) 0.5 mg/mL (Merck, Darmstadt, Germany) and processed as elsewhere reported [18]. The optical density in each well was measured using a spectrophotometer (Thermo Fisher Scientific, Waltham, MA, USA) at a wavelength of 540 nm. Each experiment was performed three times in triplicates per experimental condition (n = 9).

#### 4.5.4. Cell Cycle Analysis

The progression of the cell cycle was assessed by flow cytometry, as already reported [24]. Briefly, after the pre-incubation with H_2_O_2_ (100 µM for 3 h) and the exposure time (48 h), approximately 0.5 × 10^5^ cells/experimental condition were fixed with cold ethanol 70% *v*/*v* and afterwards stained with 1 mg/mL propidium iodide (PI) (final concentration 10 µg/mL) and 10 mg/mL RNAse (final concentration 100 µg/mL) and kept overnight at 4 ˚C in the dark. Each experiment was performed two times in triplicates per experimental condition (n = 6). Cell cycle profiles (1 × 10^4^ events/sample) were finally analysed with a CytoFLEX flow cytometer (Beckman Coulter, Indianapolis, IN, USA), and data were quantified using the ModFit LT™ 4.0 software (De Novo Software, Glendale, CA, USA).

#### 4.5.5. Collagen Type I Secretion

Amounts of collagen type I secreted in supernatants harvested from 96 well plates were detected by a human collagen type 1 ELISA kit (Cosmo Bio Co., Ltd., Tokyo, Japan; cat. no. ACE-EC1-E105-EX). Samples were pipetted into suitably coated wells as described elsewhere [43]. Each ELISA test was performed three times in duplicates per experimental condition (n = 6). The concentration of collagen type I (µg/mL) was calculated using a standard curve generated with a specific standard provided by the manufacturer by means of the Prism 5.0 software (GraphPad, San Diego, CA, USA).

#### 4.5.6. Statistical Analyses

Statistics for cell biological parameters were performed using one-way analysis of variance (ANOVA) followed by Dunnet and Tukey’s multiple comparison tests by means of the Prism 8.0 software (GraphPad, San Diego, CA, USA). Results are presented as mean values ± standard deviations. Values of *p* ≤ 0.05 were considered statistically significant.

## Figures and Tables

**Table 1:** Inhibition data of human CA I, CA II, CA IX and CA XII with compounds **1**–**3** (**a**–**c**) and the standard sulfonamide inhibitor acetazolamide (**AAZ**) by a stopped-flow CO_2_ hydrase assay [33].

				*K*_I_ (nM) *			
		X	R	hCA I	hCA II	hCA IX	hCA XII
**1a**	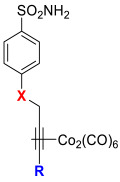	**N**	H	900.9 ^a^	215.4	10,000	10,000
**2a**			Me	957.6	2349	27.8	4.6
**3a**			Et	3010	4245	26.5	2.1
**1b**		**O**	H	1345 ^a^	40.9	5.4	0.9
**2b**			Me	560.5	7.0	19.3	93.5
**3b**			Et	930.3	600.7	29.1	3.8
**1c**	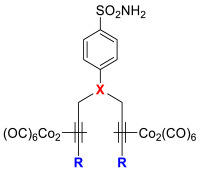	**N**	H	876 ^a^	165	6.2	7.5
**2c**			Me	6191	9.5	25.8	27.8
**3c**			Et	716.7	9.5	47.5	93.1
**AAZ**				250	12	25.8	5.7

* Mean from three different assays using a stopped-flow technique (errors were in the range of ±5–10% of the reported values). ^a^ Data from ref [22].

## Data Availability

The original contributions presented in this study are included in the article/Supplementary Material. Further inquiries can be directed to the corresponding author.

## Supplementary Materials

The following supporting information can be downloaded at: https://www.mdpi.com/article/10.3390/molecules30030593/s1: synthetic procedures for the preparation of the CORM precursors, inhibition data of the CORM precursors against hCAs, analysis of the CO units released by CORMs, ^1^H and ^13^C NMR spectra. Ref [44] is cited here.

## Abbreviations

CAI(s), carbonic anhydrase inhibitor(s); AAZ, acetazolamide; (h)CA, (human) carbonic anhydrase; CO, Carbon monoxide; CORMs, CO releasing molecules; HO, Heme Oxygenases; RA, Rheumatoid Arthritis; DCH, dicobalthexacarbonyl; Mb, Myoglobin.
