# Supplementary material for: In Vitro CO-Releasing and Antioxidant Properties of Sulfonamide-Based CAI-CORMs in a H2O2-Stimulated Human Achilles Tendon-Derived Cell Model
Source: Molecules. 2025 Jan 28;30(3):593. doi: 10.3390/molecules30030593 (PMC11819963; doi:10.3390/molecules30030593)
Supplement: Supplementary file 1 [file molecules-30-00593-s001.zip › molecules-3396486-supplementary.pdf]

Supplementary Information for

In vitro CO-Releasing and Antioxidant Properties of Sulfonamide-Based CAI-CORMs in a H_2O_2-Stimulated Human Achilles Tendon-Derived Cell Model

Emanuela Berrino,[1] Paolo Guglielmi,[1,*] Fabrizio Carta,[2] Simone Carradori,[3] Cristina Campestre,[3] Andrea Angeli,[2] Francesca Arrighi,[1] Virginia Pontecorvi,[1] Paola Chimenti,[1] Daniela Secci,[1] Claudiu T. Supuran,[2] Marialucia Gallorini[3]

[1]Department of Drug Chemistry and Technologies, Sapienza University of Rome, P.le A. Moro 5, 00185 Rome, Italy

[2]NEUROFARBA Department, Sezione di Scienze Farmaceutiche e Nutraceutiche, University of Florence, 50019, Sesto Fiorentino, Florence, Italy

[3]Department of Pharmacy, "G. d'Annunzio" University of Chieti-Pescara, via dei Vestini 31, 66100 Chieti, Italy

*Corresponding author: Dr. Paolo Guglielmi

Table of contents

**Synthetic procedures for the preparation of the CORM precursors**	**S2-S5**
**Inhibition data of the CORM precursors against hCAs**	**S6**
**Analysis of the CO units released by CORMs**	**S7**
**^{1}H and ^{13}C NMR spectra**	**S8-S26**
**References**	**S27**

General procedure 1 for the preparation of alkyne precursors A, A-II, A-III and C, C-II, C-III

To a solution of sulfanilamide (0.5 g, 1.0 eq.) in dry DMF (2 ml) and pyridine (1.2 eq.), the appropriate alkyne (1.5 eq.) was added dropwise under N_2 atmosphere and the mixture was stirred at 70 °C o.n. (TLC monitoring). The reaction was quenched with slush and extracted with EtOAc (3 x 15 ml). The combined organic layers were washed with H_2O (3 x 15 ml) and brine (3 x 15 ml), dried over anhydrous Na_2SO_4, filtered-off and concentrated under vacuum to give a solid that was purified by silica gel column chromatography, eluting with the appropriate mixture of EtOAc in *n*-Hexane to isolate the desired compounds.

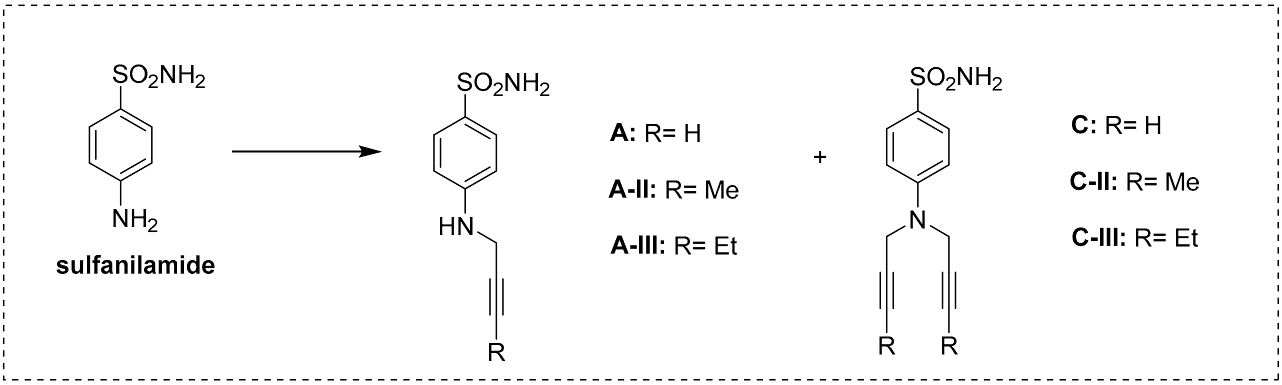

General procedure 2 for the preparation of alkyne precursors B, B-II, B-III, pre-CO-A, pre-CO-B, pre-CO-C

To a solution of 4-hydroxybenzensulfonamide or aniline or phenol (0.5 g, 1.0 eq.) in dry DMF (4 mL) under N_2 atmosphere, K_2CO_3 (2.0 eq.) was added. After 10 min, the appropriate alkyne (1.2 or 1.5 eq.) was added dropwise and the mixture stirred at 60 °C until consumption of starting material (5 h, TLC monitoring). The reaction was quenched with slush and extracted with EtOAc (3 x 15 ml). The combined organic layers were washed with H_2O (3 x 15 ml) and brine (3 x 15 ml), dried over anhydrous Na_2SO_4, filtered-off and concentrated under vacuum to give a solid that was purified by silica gel column chromatography, eluting with the appropriate mixture of EtOAc in *n*-Hexane to afford the desired products.

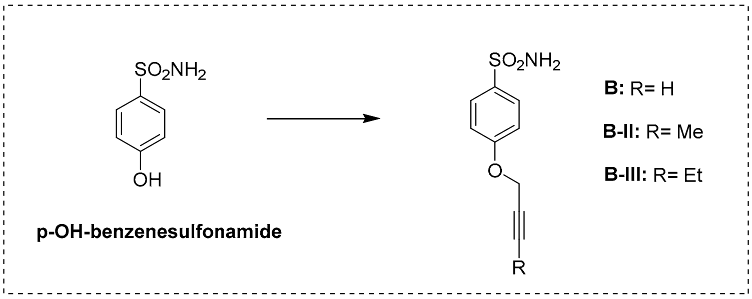

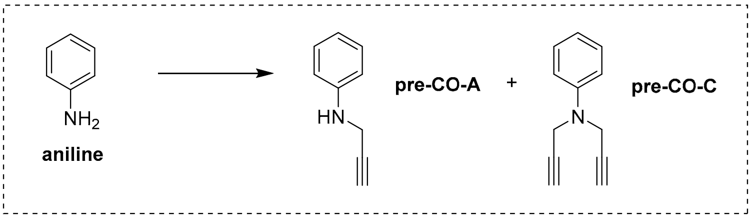

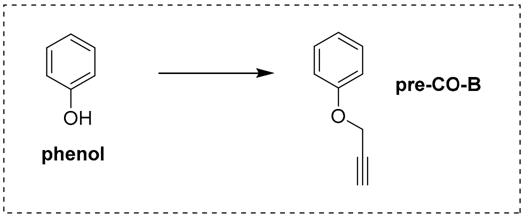

4-(Prop-2-ynylamino)benzenesulfonamide (A) and 4-(di(prop-2-yn-1-yl)amino)benzenesulfonamide (C). The titled compounds **A** and **C** were obtained according to the general procedure **1** using propargyl bromide 80% in toluene as alkyne. Purified by silica gel column chromatography eluting with 50% ethyl acetate in *n*-hexane to separate and isolate the desired products **A-I** and **C-I** as yellow solids. **4-(Prop-2-ynylamino)benzenesulfonamide (A):** 33 % yield; silica gel TLC R_f 0.30 (EtOAc/n-Hex 30 % *v/v*); δ_H (400 MHz, DMSO-d_6): 3.10 (1H, t, J = 2.4, C*H*), 3.93 (2H, dd, J= 2.4, 6.0, CH_2), 6.69 (2H, d, J = 8.8, Ar-*H*), 6.72 (1H, br t, exchange with D_2O, N*H*), 6.95 (2H, br.s, exchange with D_2O, SO_2NH_2), 7.55 (2H, d, J = 8.8, Ar-*H*). **4-(di(prop-2-yn-1-yl)amino)benzenesulfonamide (C)**: 25 % yield; silica gel TLC R_f 0.56 (EtOAc/n-Hex 30 % *v/v*); δ_H (400 MHz, DMSO-d_6): 3.18 (2H, t, J = 2.4, 2 x C*H*), 4.25 (4H, d, J = 2.4, 2 x CH_2), 6.99 (2H, d, J = 8.8, Ar-*H*), 7.05 (2H, s, exchange with D_2O, SO_2NH_2), 7.67 (2H, d, J = 8.8, Ar-*H*). Experimental results in agreement with reported data.[1]

4-(but-2-yn-1-ylamino)benzenesulfonamide (A-II) and 4-(di(but-2-yn-1-yl)amino)benzenesulfonamide (C-II). The titled compounds **A-II and C-II** were obtained according to the general procedure **1** using 1-bromobut-2-yne as alkyne. Purified by silica gel column chromatography eluting with 40% ethyl acetate in *n*-hexane to separate and isolate the desired products **A-II** and **C-II** as white solids. **4-(but-2-yn-1-ylamino)benzenesulfonamide (A-II):** 20 % yield; silica gel TLC R_f 0.40 (EtOAc/n-Hex 50 % *v/v*); δ_H (400 MHz, DMSO-d_6): 1.76 (3H, t, J = 2.5, CH_3), 3.88 (2H, m, CH_2), 6.66 (2H, d, J = 8.7, Ar-*H*), 6.69 (1H, br t, exchange with D_2O, N*H*), 6.96 (2H, br.s, exchange with D_2O, SO_2NH_2), 7.54 (2H, d, J = 8.8, Ar-*H*); δ_C (100 MHz, DMSO-d_6): 3.0, 31.9, 76.5, 78.1, 111.4, 127.2, 130.0, 150.6. **4-(di(but-2-yn-1-yl)amino)benzenesulfonamide (C-II):** 14% yield; silica gel TLC R_f 0.23 (EtOAc/n-Hex 30 % *v/v*); δ_H (400 MHz, DMSO-d_6): 1.76 (6H, t, J = 2.3, 2 x CH_3), 4.17 (4H, q, J = 2.4, 2 x CH_2), 6.94 (2H, d, J = 9.0, Ar-*H*), 7.05 (2H, br.s, exchange

with D_2O, SO_2NH_2), 7.64 (2H, d, *J* = 8.8, Ar-*H*); δ_C (100 MHz, DMSO-*d*$_6$): 3.1, 40.0, 74.8, 79.9, 113.1, 127.0, 132.4, 149.6.

4-(pent-2-yn-1-ylamino)benzenesulfonamide (A-III) and 4-(di(pent-2-yn-1-yl)amino)benzenesulfonamide (C-III). The titled compounds **A-III** and **C-III** were obtained according to the general procedure **1** using 1-bromopent-2-yne as alkyne. Purified by silica gel column chromatography eluting with 40% ethyl acetate in *n*-hexane to separate and isolate the desired products **A-III** and **C-III** as white solids. **4-(pent-2-yn-1-ylamino)benzenesulfonamide (A-III):** 22% yield; silica gel TLC R_f 0.42 (EtOAc/n-Hex 50 % *v/v*); δ_H (400 MHz, DMSO-*d*$_6$): 1.03 (3H, t, *J* = 7.5, C*H*$_3$), 2.15 (2H, m, C*H*$_2$), 3.89 (2H, dt, *J* = 2.3, 4.9, C*H*$_2$), 6.67 (3H, m, 2 x Ar-*H*, 1 x N*H*, exchange with D_2O), 6.95 (2H, br.s, exchange with D_2O, SO_2NH_2), 7.53 (2H, d, *J* = 8.6, Ar-*H*); δ_C (100 MHz, DMSO-*d*$_6$): 11.6, 13.8, 32.0, 76.7, 83.9, 111.4, 127.2, 130.9, 150.6. **4-(di(pent-2-yn-1-yl)amino)benzenesulfonamide (C-III)**: 13% yield; silica gel TLC R_f 0.25 (EtOAc/n-Hex 30 % *v/v*); δ_H (400 MHz, DMSO-*d*$_6$): 1.02 (6H, t, *J* = 7.5, 2 x C*H*$_3$), 2.14 (4H, qt, *J* = 7.4, 2.2, 2 x C*H*$_2$), 4.19 (4H, t, *J* = 2.3, 2 x C*H*$_2$), 6.94 (2H, d, *J* = 8.8, Ar-*H*), 7.04 (2H, br.s, exchange with D_2O, SO_2NH_2), 7.63 (2H, d, *J* = 8.7, Ar-*H*); δ_C (100 MHz, DMSO-*d*$_6$): 11.6, 13.7, 39.5 (overlapped with DMSO signal), 74.9, 85.5, 113.1, 126.9, 132.4, 149.5.

4-(Prop-2-ynyloxy)benzenesulfonamide (B). The titled compound **B-I** was obtained according to the general procedure **2** using propargyl bromide 80% in toluene as alkyne. Purified by silica gel column chromatography eluting with 40% ethyl acetate in *n*-hexane to afford the desired product **B-I** as a white powder. 53% yield; silica gel TLC R_f 0.28 (EtOAc/n-Hex 40 % *v/v*); δ_H (400 MHz, DMSO-*d*$_6$) 3.61 (1H, br s, C*H*), 4.90 (2H, br s, C*H*$_2$), 7.13 (2H, d, *J* = 7.2, Ar-*H*), 7.23 (2H, s, exchange with D_2O, SO_2NH_2), 7.77 (2H, d, *J* = 7.2, Ar-*H*). Experimental results were in agreement with reported data.[2]

4-(but-2-yn-1-yloxy)benzenesulfonamide (B-II). The titled compound **B-II** was obtained according to the general procedure **2** using 1-bromobut-2-yne as alkyne. Purified by silica gel column chromatography eluting with 40% ethyl acetate in *n*-hexane to afford the desired product **B-II** as a white powder. 46% yield; silica gel TLC R_f 0.27 (EtOAc/n-Hex 40 % *v/v*); δ_H (400 MHz, DMSO-d$_6$) 1.83 (3H, t, *J* = 2.3, C*H*$_3$), 4.83 (2H, q, *J* = 2.4, C*H*$_2$), 7.11 (2H, d, *J* = 8.9, Ar-*H*), 7.21 (2H, s, exchange with D_2O, SO_2NH_2), 7.75 (2H, d, *J* = 8.8, Ar-*H*); δ_C (100 MHz, DMSO-*d*$_6$): 3.1, 56.2, 74.1, 84.1, 114.8, 127.6, 136.6, 159.7.

4-(pent-2-yn-1-yloxy)benzenesulfonamide (B-III). The titled compound **B-III** was obtained according to the general procedure **2** using 1-bromopent-2-yne as alkyne. Purified by silica gel column chromatography eluting with 30% ethyl acetate in *n*-hexane to afford the desired product **B-III** as a white powder. 43% yield; silica gel TLC R*f* 0.37 (EtOAc/n-Hex 40% *v/v*); δ_H (400 MHz,

DMSO-d_6) 1.05 (3H, t, *J* = 7.4, C*H*$_3$), 2.23 (2H, qt, *J* = 2.2, 7.5, C*H*$_2$), 4.84 (2H, q, *J* = 2.2, C*H*$_2$), 7.11 (2H, d, *J* = 8.8, Ar-*H*), 7.21 (2H, s, exchange with D_2O, SO_2NH_2), 7.75 (2H, d, *J* = 8.8, Ar-*H*); δ_C (100 MHz, DMSO-d_6): 11.5, 13.3, 56.1, 74.2, 89.4, 114.7, 127.4, 136.5, 159.7.

***N*-(prop-2-yn-1-yl)aniline pre-CO-A and *N,N*-di(prop-2-yn-1-yl)aniline pre-CO-C.** The titled compounds **pre-CO-A** and **pre-CO-C** were obtained according to the general procedure **2** using propargyl bromide 80% in toluene as alkyne. Purified by silica gel column chromatography eluting with 10% ethyl acetate in *n*-hexane to separate and isolate the desired products **pre-CO-A** and **pre-CO-C**. ***N*-(prop-2-yn-1-yl)aniline pre-CO-A:** 20% yield; yellow solid; silica gel TLC R*f* =0.45 (EtOAc/n- Hex 10% v/v); δ_H (400 MHz, $CDCl_3$): 2.25 (1H, t, *J* = 2.7, C*H*), 3.78 (1H, br t, exchange with D_2O, N*H*), 3.94 (2H, t, *J*= 2.5, C*H*$_2$), 6.72 (2H, d, *J* = 8.1, Ar-*H*), 6.84 (1H, t, *J* = 7.4, Ar-*H*), 7.26 (2H, t, *J* = 8.1, Ar-*H*). Experimental results are in agreement with reported data.[2]

***N,N*-di(prop-2-yn-1-yl)aniline pre-CO-C**: 11% yield; yellow oil. silica gel TLC R*f*=0.59 (EtOAc/n-Hex 10% v/v); δ_H (400 MHz, $CDCl_3$): 2.28 (2H, t, *J* = 2.4, 2 x C*H*), 4.15 (4H, d, *J*= 2.3, 2 x C*H*$_2$), 6.93 (1H, t, *J* = 7.5, Ar-*H*), 7.00 (2H, d, *J* = 8.2, Ar-*H*), 7.33 (2H, t, *J* = 7.8, Ar-*H*). Experimental results are in agreement with reported data.[2]

(prop-2-yn-1-yloxy)benzene pre-CO-B. The titled compound **pre-CO-B** was obtained according to the general procedure **2** using propargyl bromide 80% in toluene as alkyne. Purified by silica gel column chromatography eluting with 30% ethyl acetate in *n*-hexane to afford the desired product **B** as colorless oil. 53% yield; δ_H (400 MHz, DMSO-d_6) 3.59 (1H, t, *J* =2.4, C*H*), 4.83 (2H, d, *J* = 2.4, C*H*$_2$), 7.01 (3H, m, Ar-*H*), 7.35 (2H, m, Ar-*H*). Experimental results are in agreement with reported data.[3]

Table S1. Inhibition data of hCA I, hCA II, hCA IX and hCA XII with compounds **1-9** and the standard sulfonamide inhibitor acetazolamide (**AAZ**) by a Stopped flow CO_2 hydrase assay. [1]

				K_I (nM)*			
		**X**	**R**	**hCA I**	**hCA II**	**hCA IX**	**hCA XII**
**A**[a]	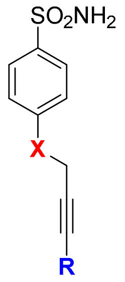	**N**	H	851.7	96.5	415.9	783.8
**A-II**			Me	54.2	2.8	214.2	0.72
**A-III**			Et	53.9	6.1	38.3	68.9
**B**[a]		**O**	H	2340	28.7	8.2	2.3
**B-II**			Me	49.7	8.3	285.9	372.3
**B-III**			Et	49.1	1.9	76.3	0.22
**C**[a]	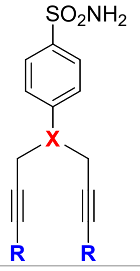	**N**	H	6400	1785	9.0	4.9
**C-II**			Me	15.7	1.9	13.6	0.56
**C-III**			Et	9.2	3.2	134.6	80.6
**AAZ**				250	12	25.8	5.7

*Mean from 3 different assays. by a stopped flow technique (errors were in the range of ± 5-10% of the reported values). [a]Data from ref [1,2]

Analysis of the CO units released by CORMs

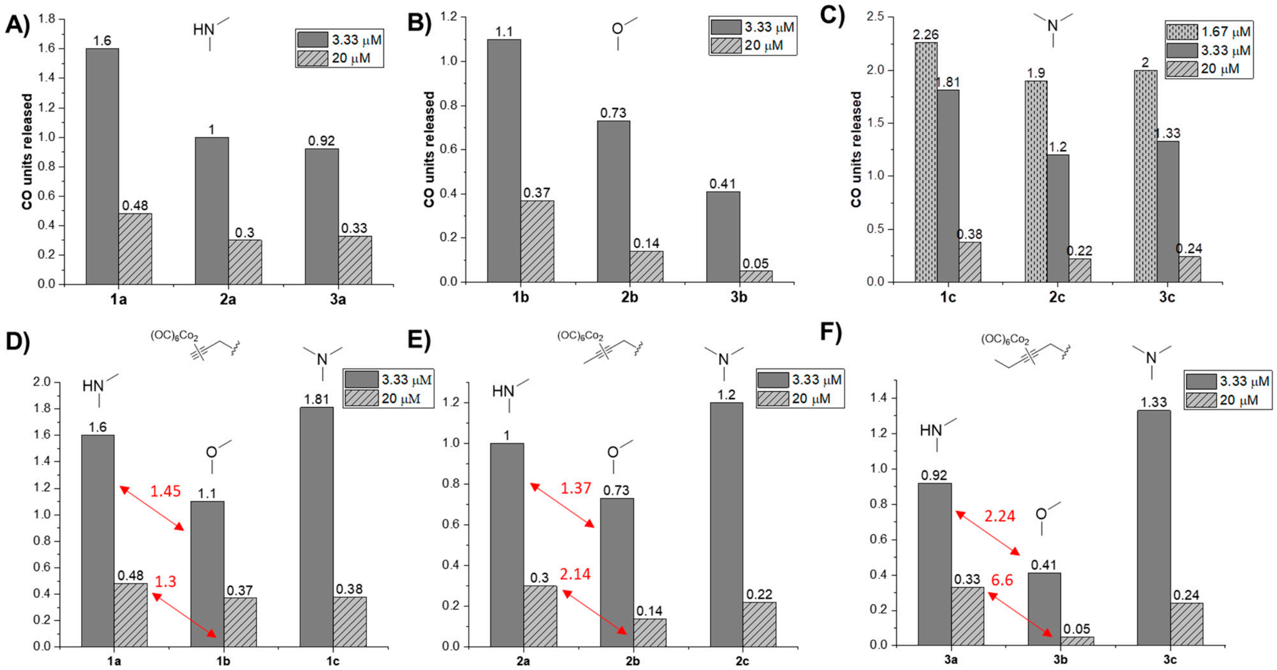

Figure S1. A) CO units released by compounds **1a**, **2a** and **3a** after 300 minutes of incubation working at 1:6 (3.33 µM CORM concentration) and 1:1 (20 µM CORM concentration) CORM-Mb ratios; B) CO units released by compounds **1b**, **2b** and **3b** after 300 minutes of incubation working at 1:6 (3.33 µM CORM concentration) and 1:1 (20 µM CORM concentration) CORM-Mb ratios; C) CO units released by compounds **1c**, **2c** and **3c** after 300 minutes of incubation working at 1:12 (1.67 µM CORM concentration), 1:6 (3.33 µM CORM concentration) and 1:1 (20 µM CORM concentration) CORM-Mb ratios; D) CO units released by compounds **1a**, **1b** and **1c** after 300 minutes of incubation working at 1:6 (3.33 µM CORM concentration) and 1:1 (20 µM CORM concentration) CORM-Mb ratios; E) CO units released by compounds **2a**, **2b** and **2c** after 300 minutes of incubation working at 1:6 (3.33 µM CORM concentration) and 1:1 (20 µM CORM concentration) CORM-Mb ratios; F) CO units released by compounds **3a**, **3b** and **3c** after 300 minutes of incubation working at 1:6 (3.33 µM CORM concentration) and 1:1 (20 µM CORM concentration) CORM-Mb ratios.

^{1}H NMR compound A

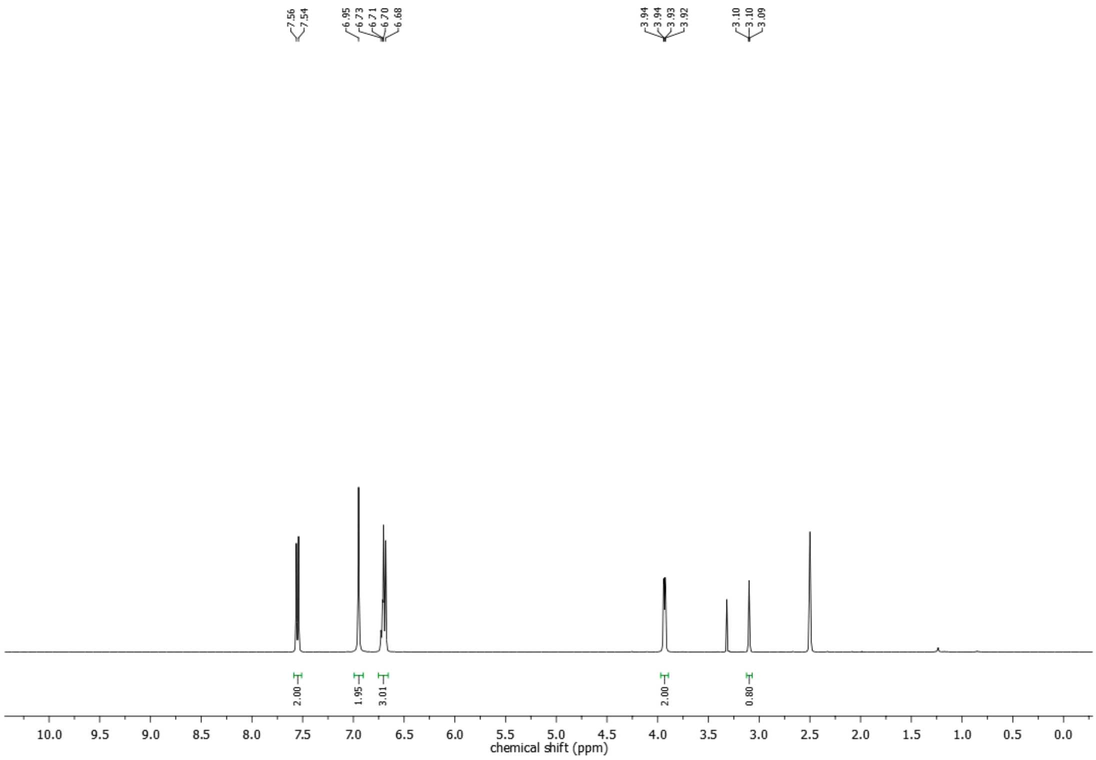

^{1}H NMR compound A-II

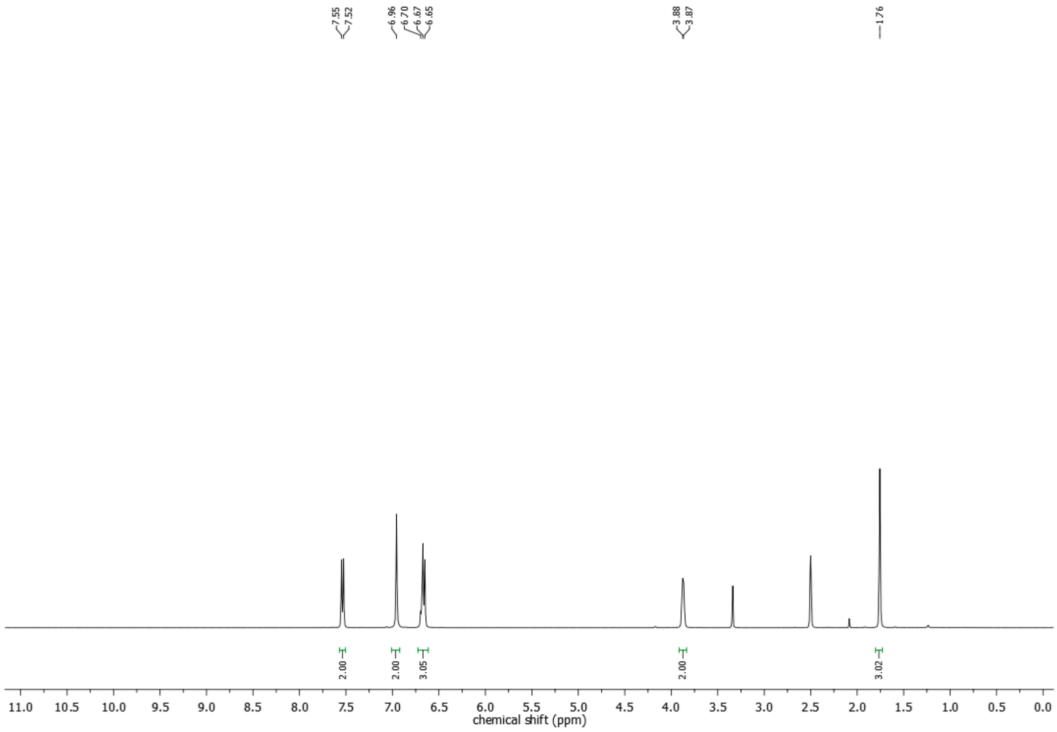

^{13}C NMR compound A-II

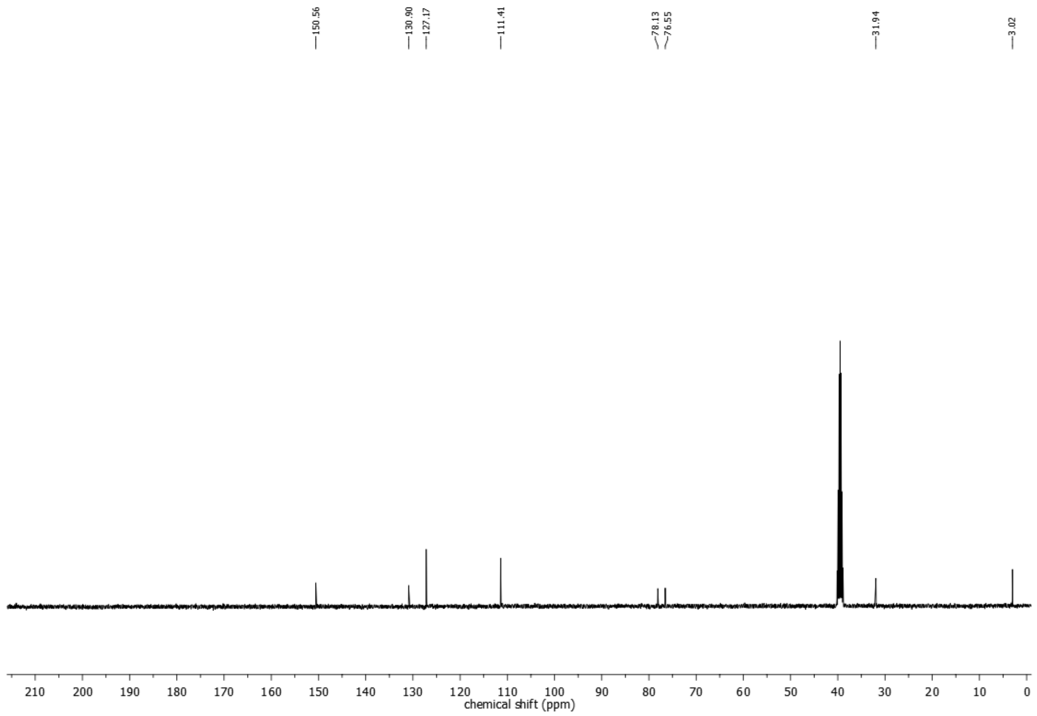

^{1}H NMR compound A-III

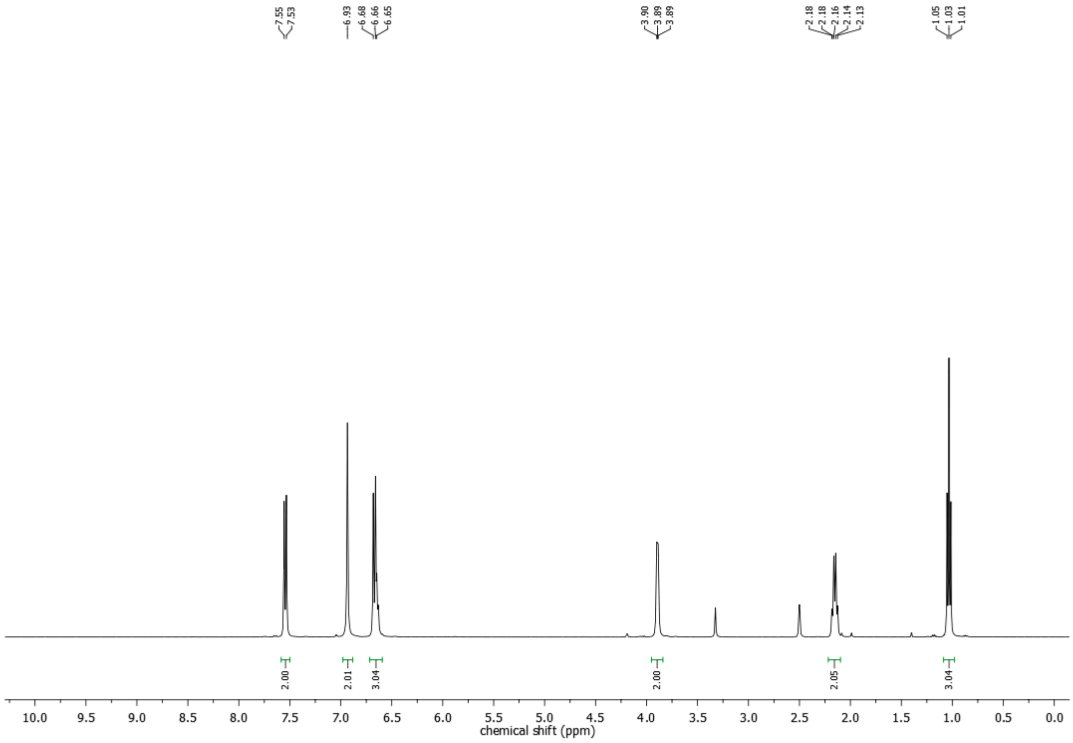

^{13}C NMR compound A-III

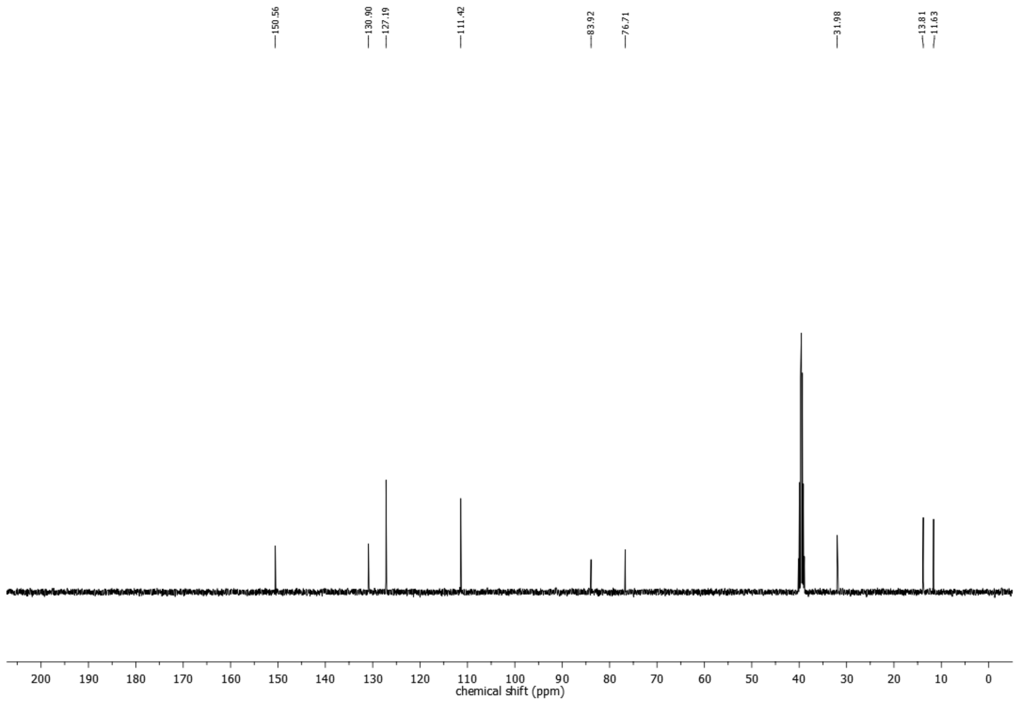

^{1}H NMR compound B

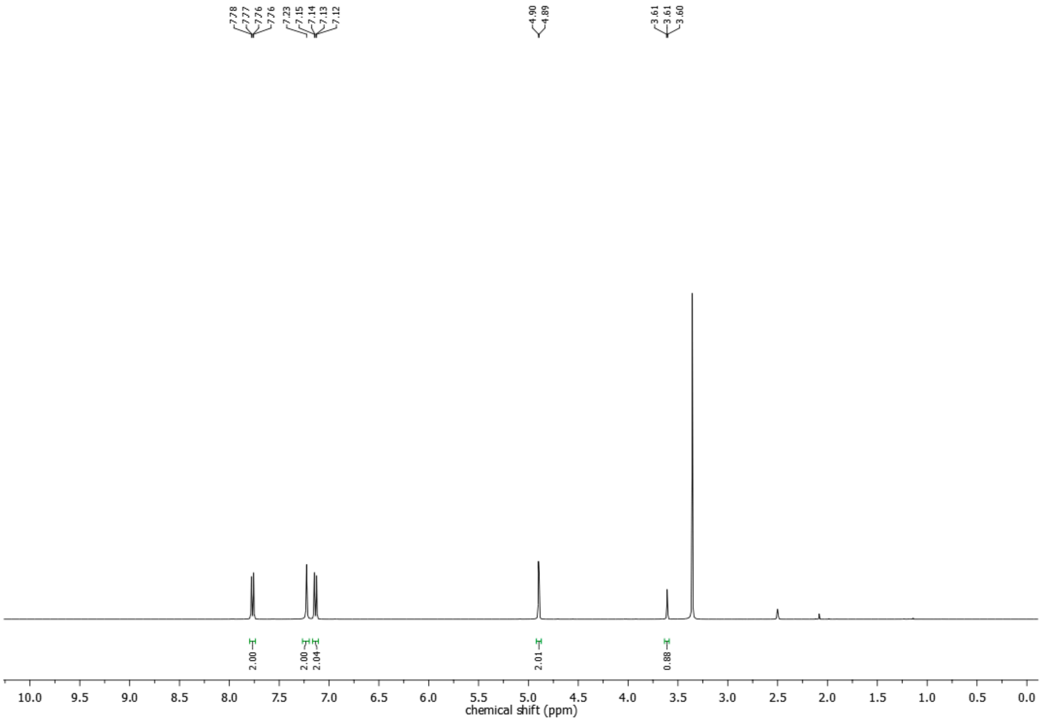

^{1}H NMR compound B-II

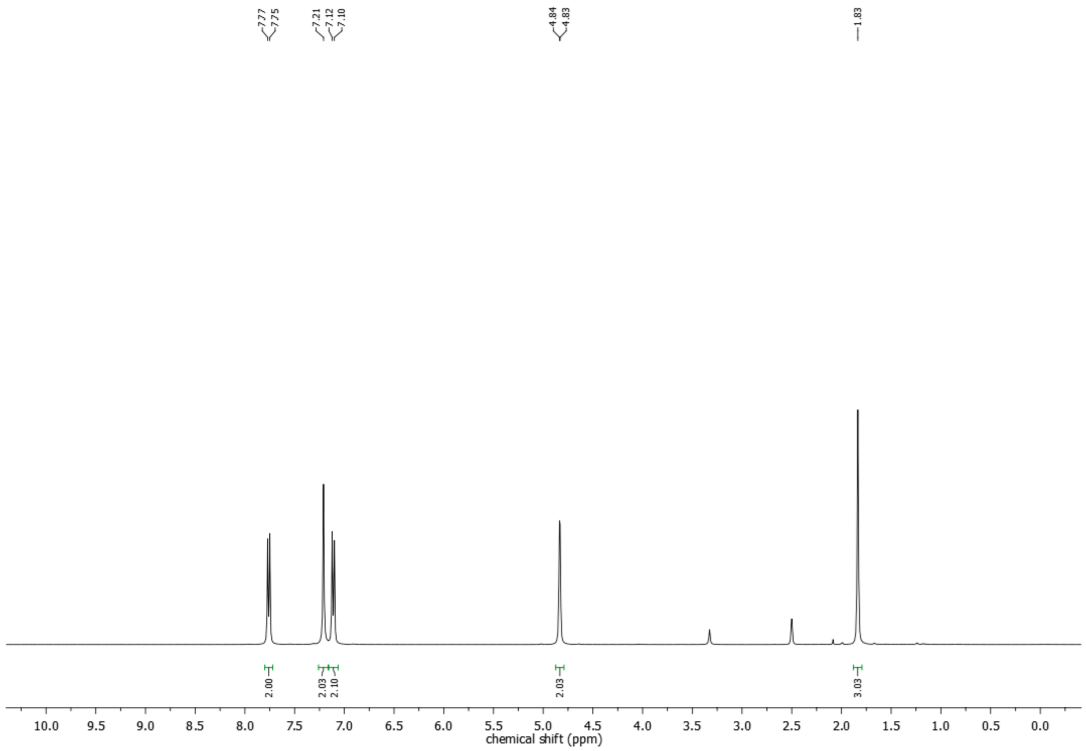

^{13}C NMR compound B-II

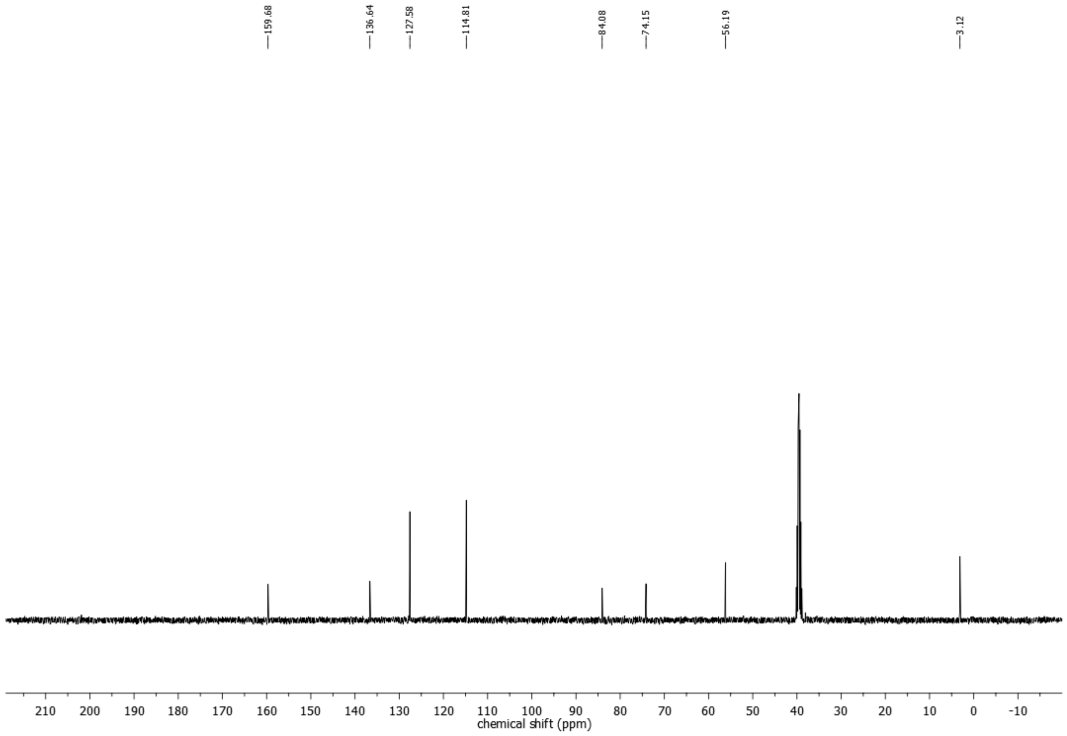

^{1}H NMR compound B-III

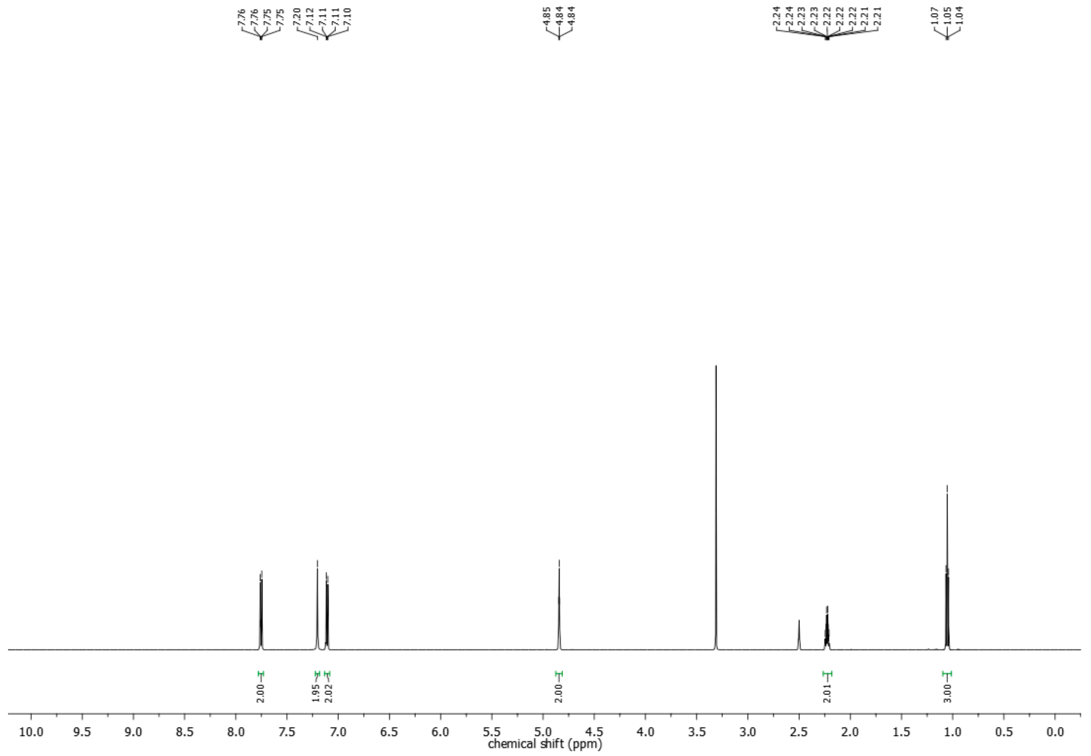

^{13}C NMR compound B-III

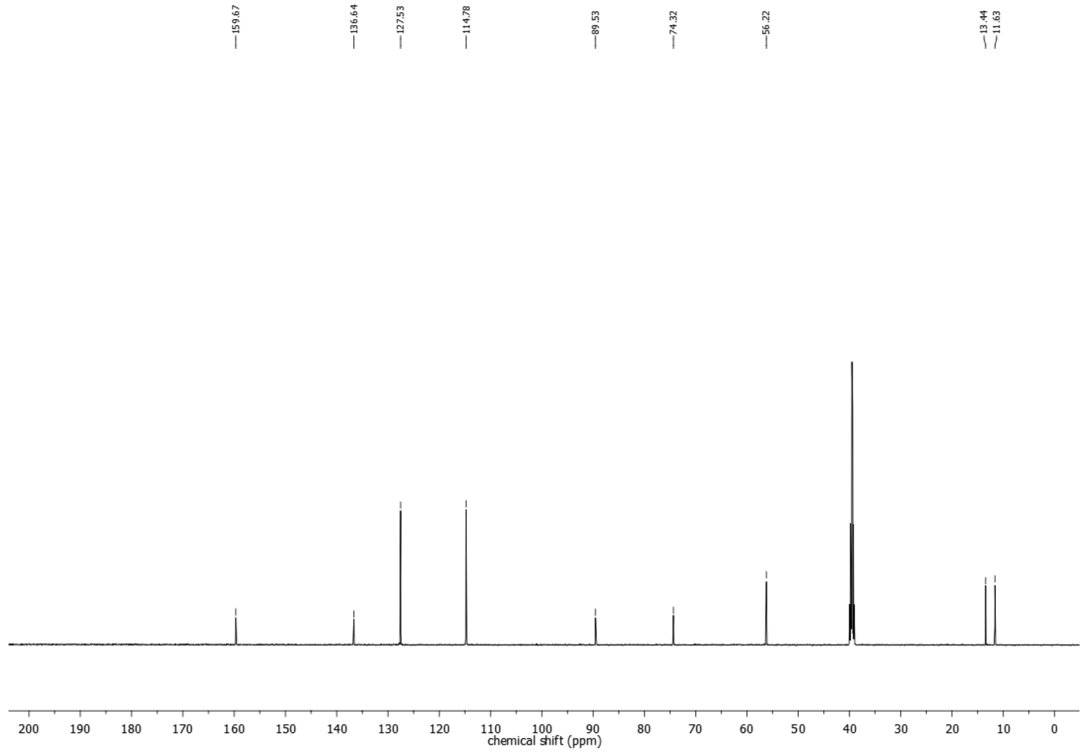

^{1}H NMR compound C

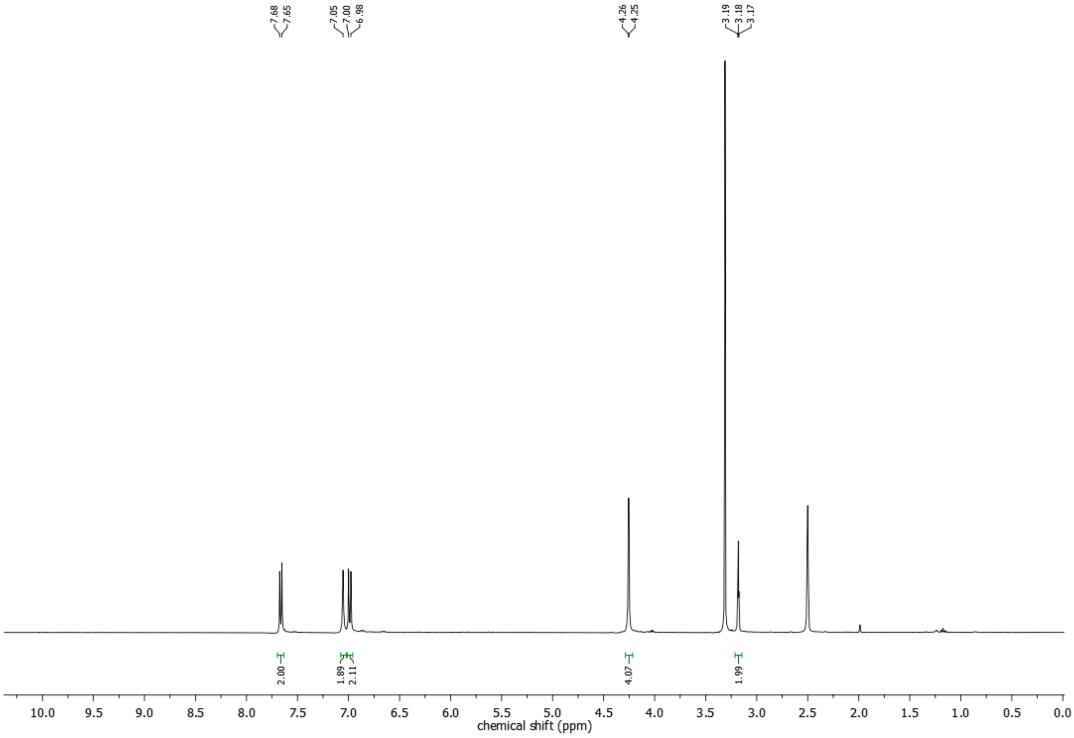

^{1}H NMR compound C-II

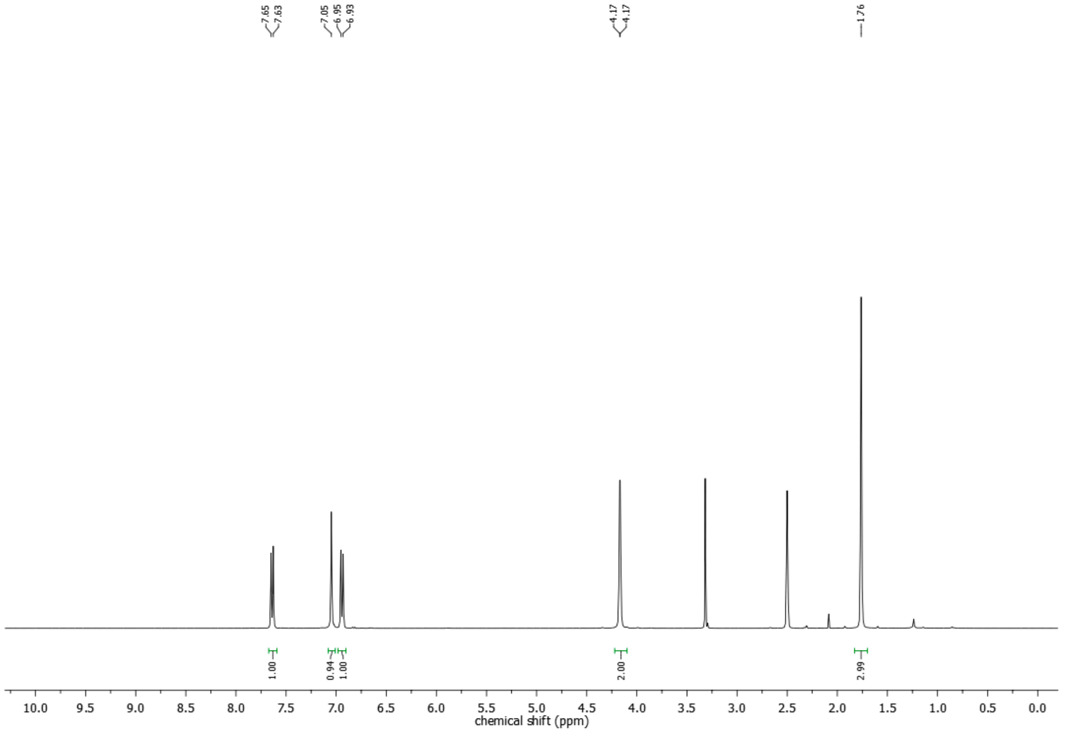

^{13}C NMR compound C-II

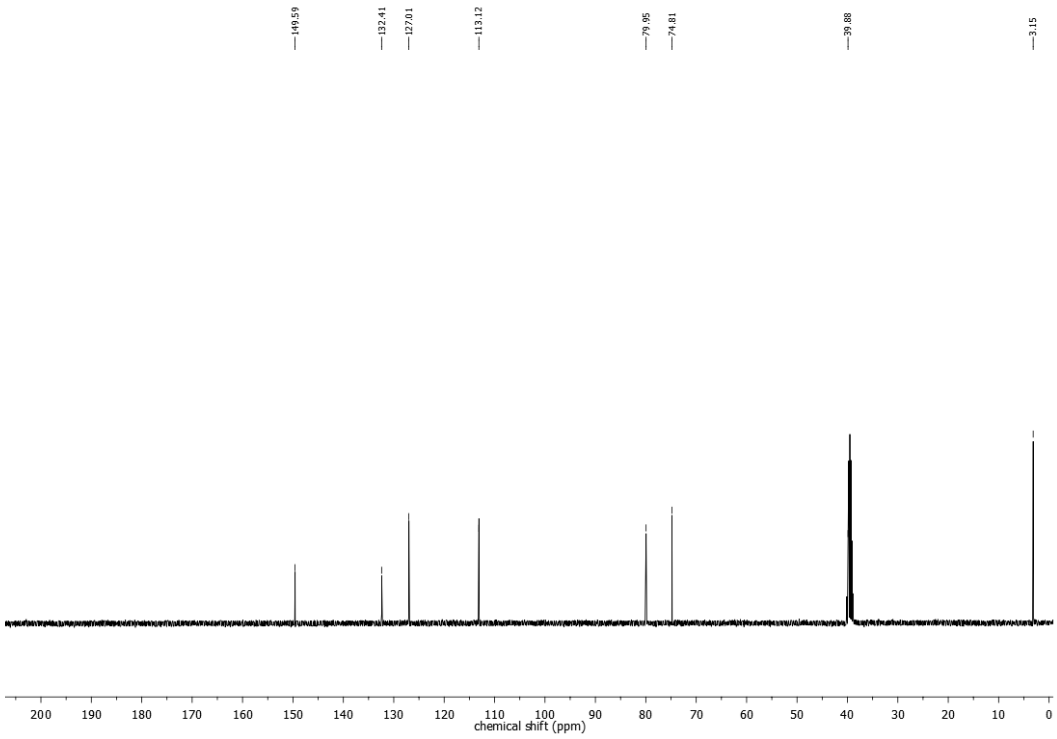

^{1}H NMR compound C-III

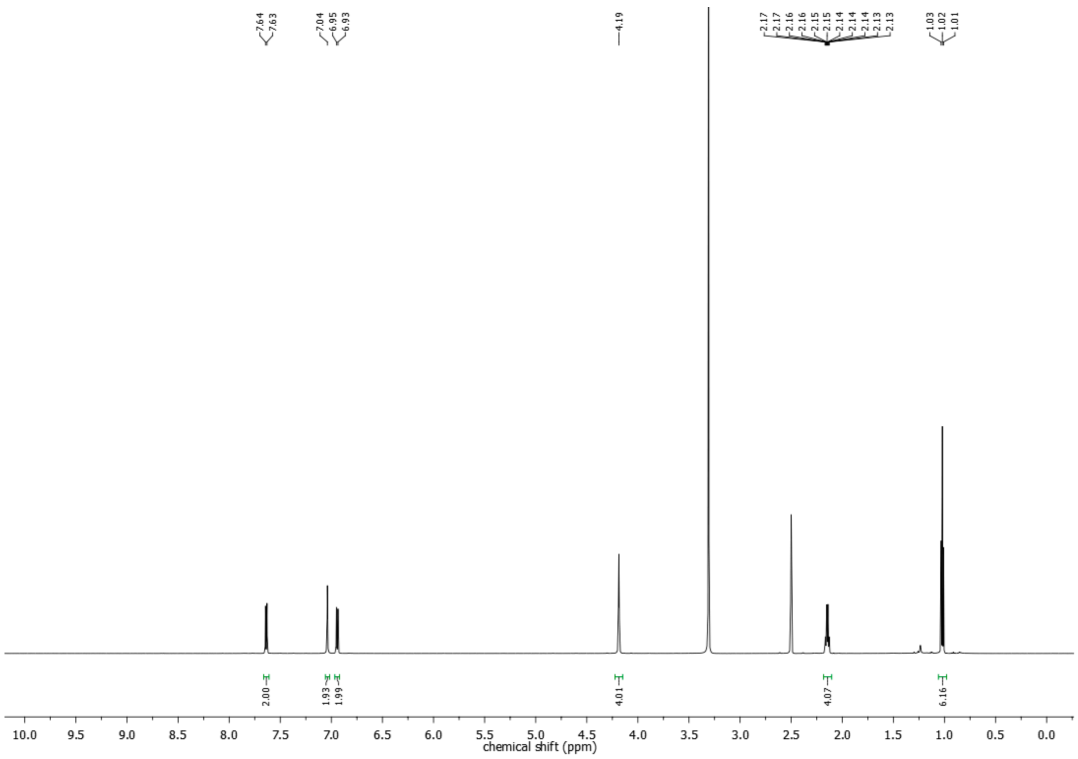

^{13}C NMR compound C-III

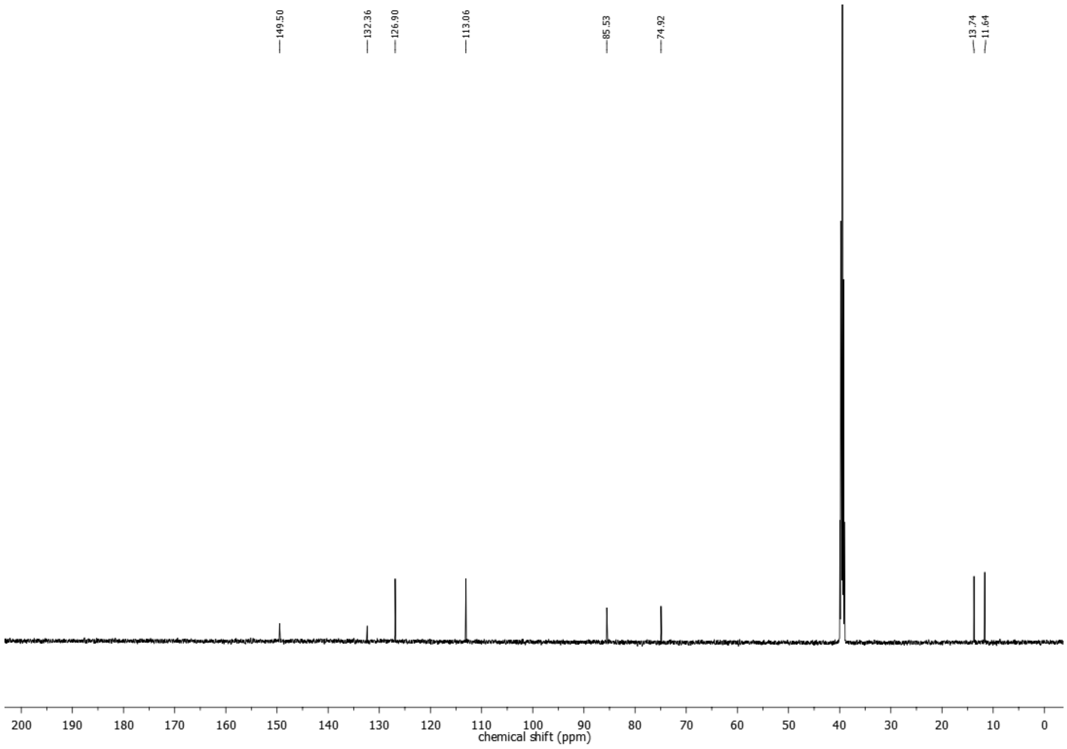

^{1}H NMR compound pre-CO-A ($CDCl_3$)

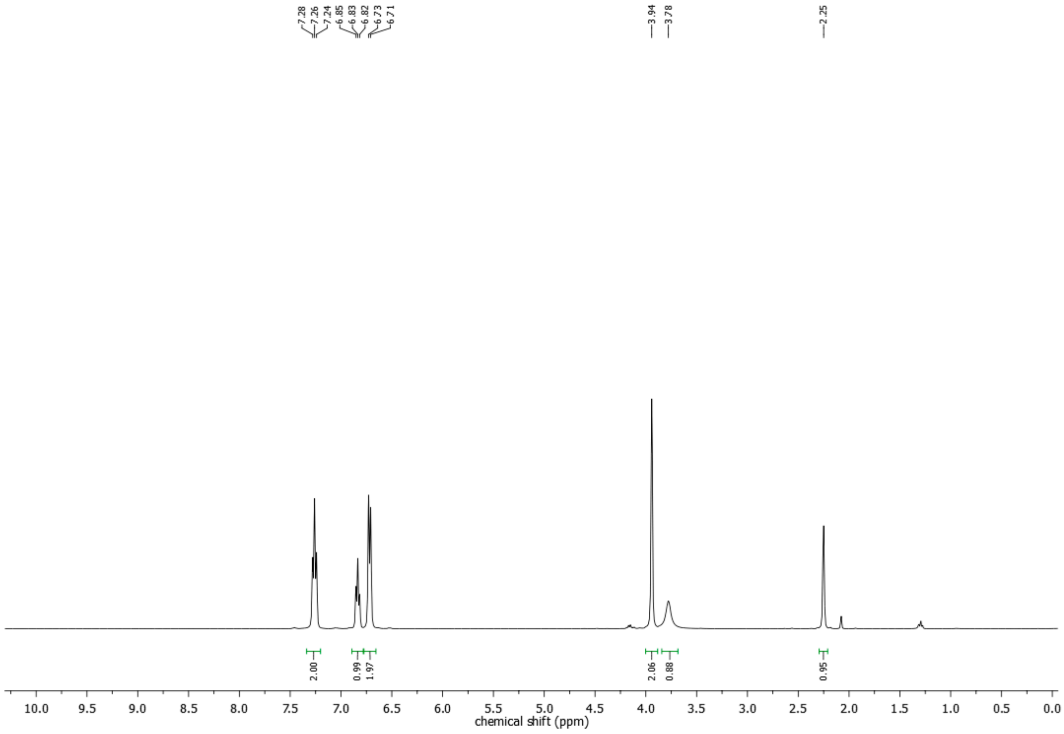

^{1}H NMR compound pre-CO-B

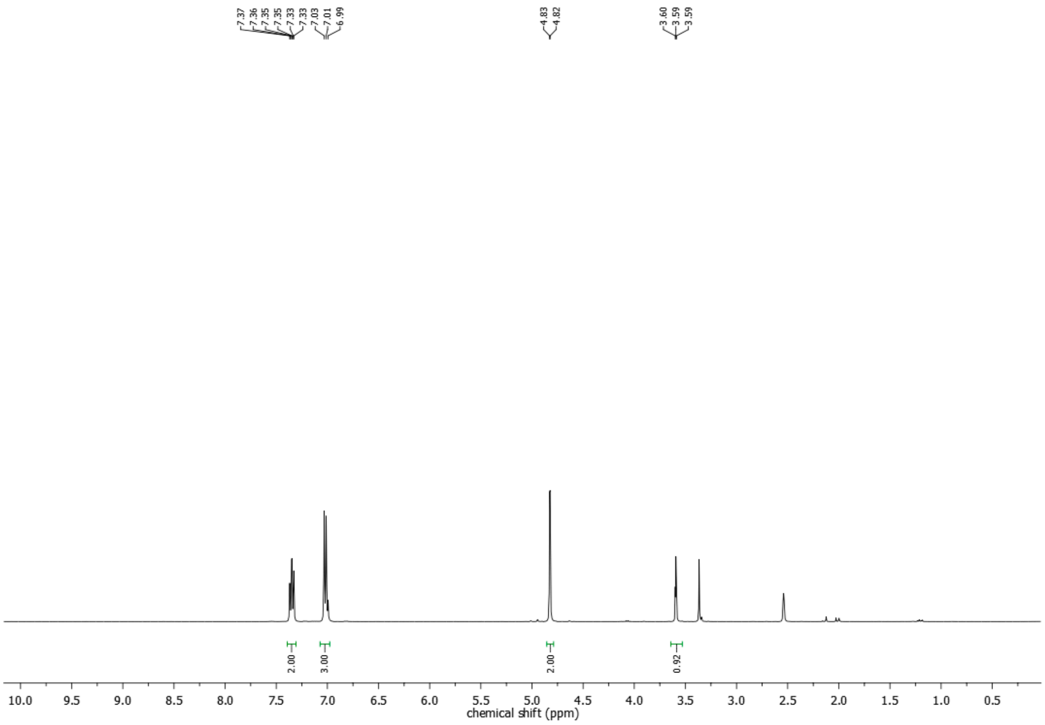

^{1}H NMR compound pre-CO-C ($CDCl_3$)

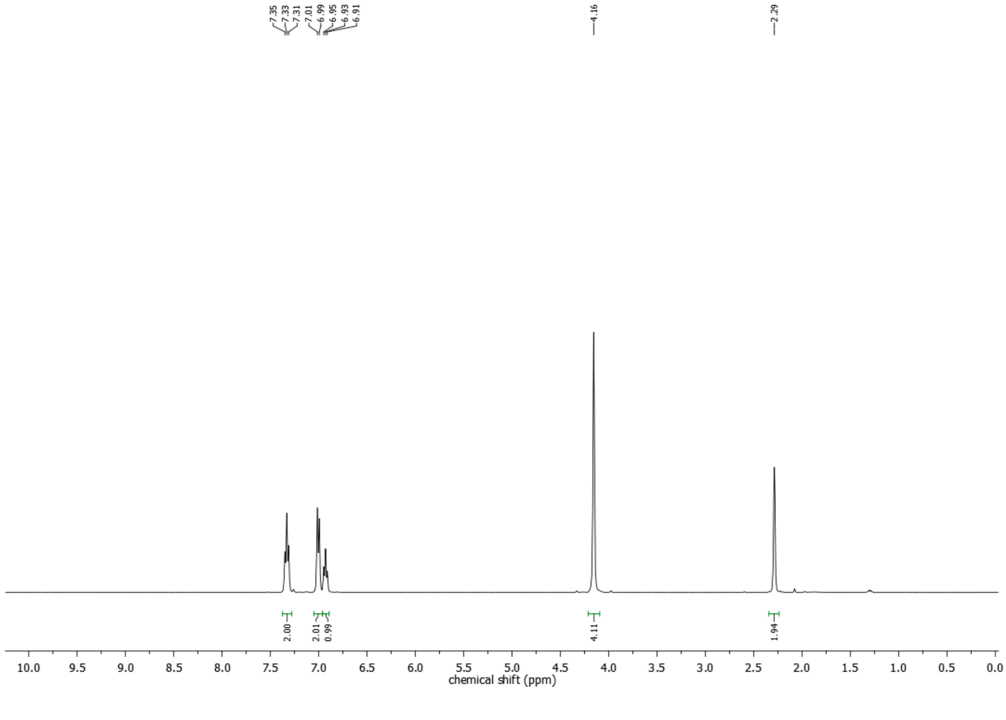

^{1}H NMR compound 1a

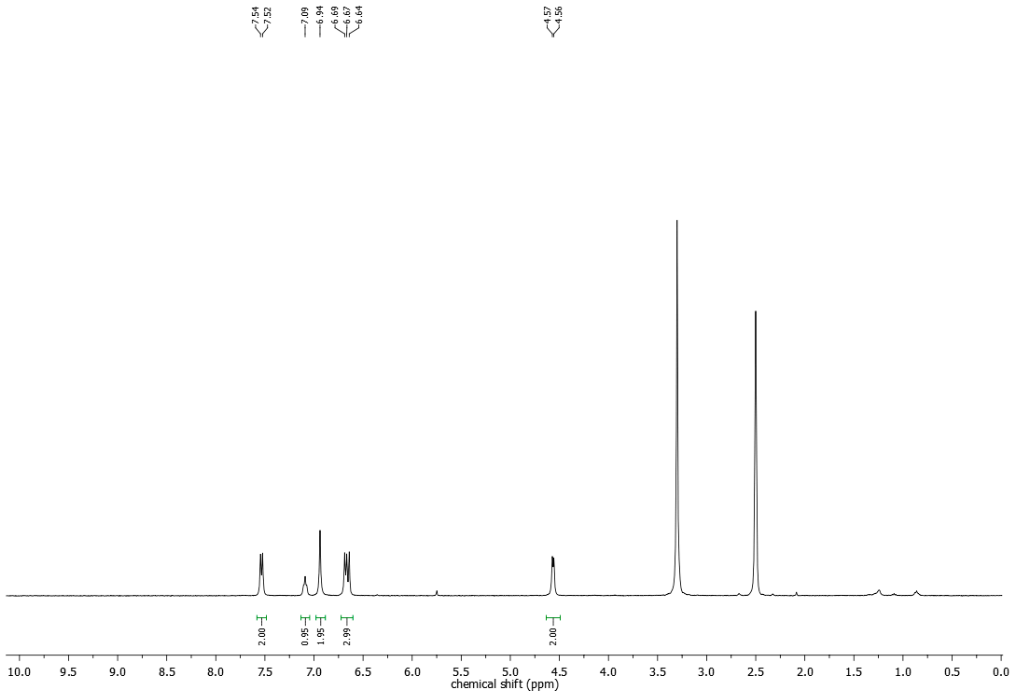

^{1}H NMR compound 2a

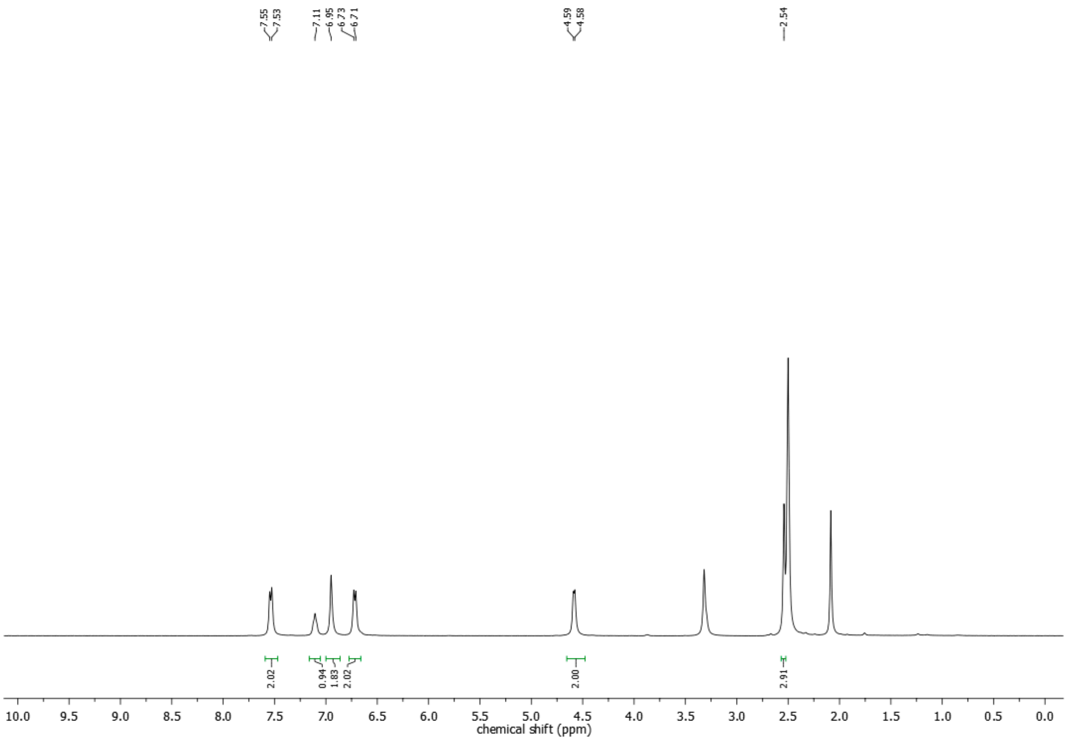

^{13}C NMR compound 2a

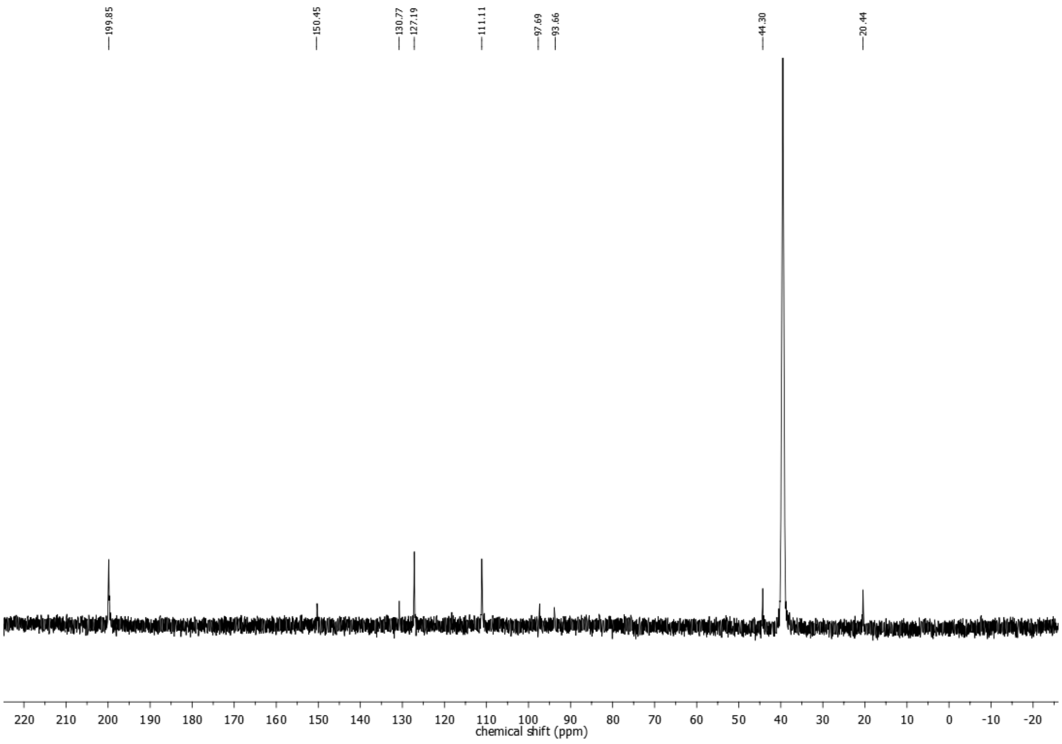

^{1}H NMR compound 3a

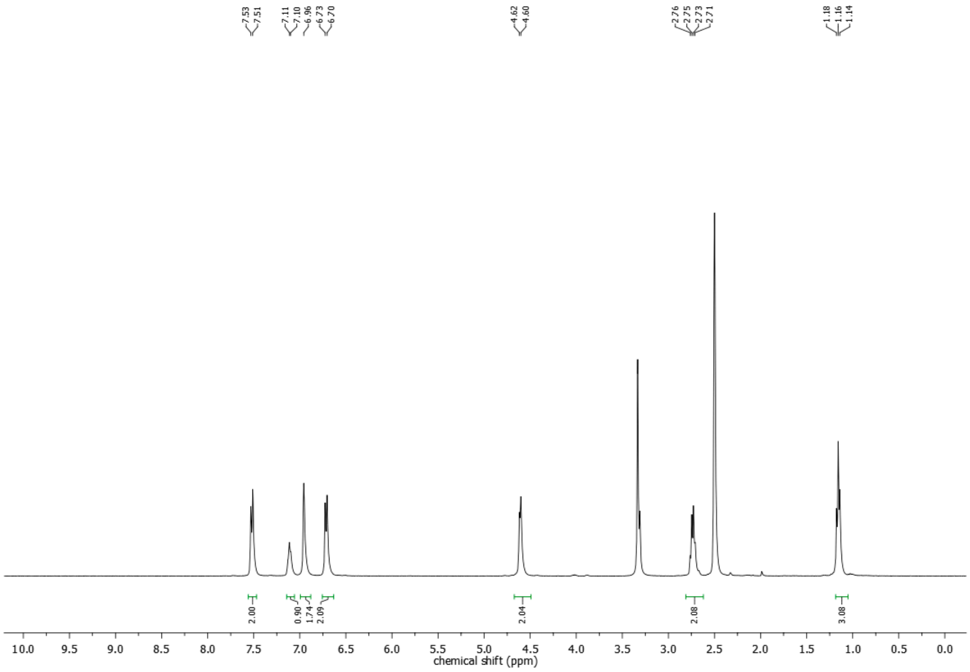

^{13}C NMR compound 3a

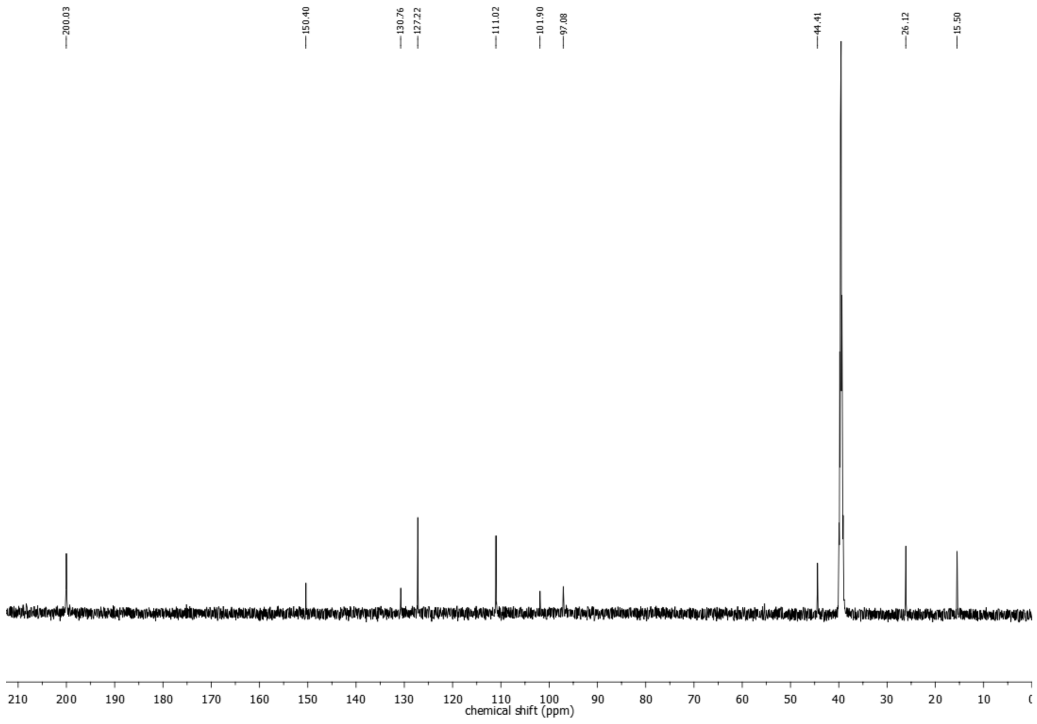

^{1}H NMR compound 1b

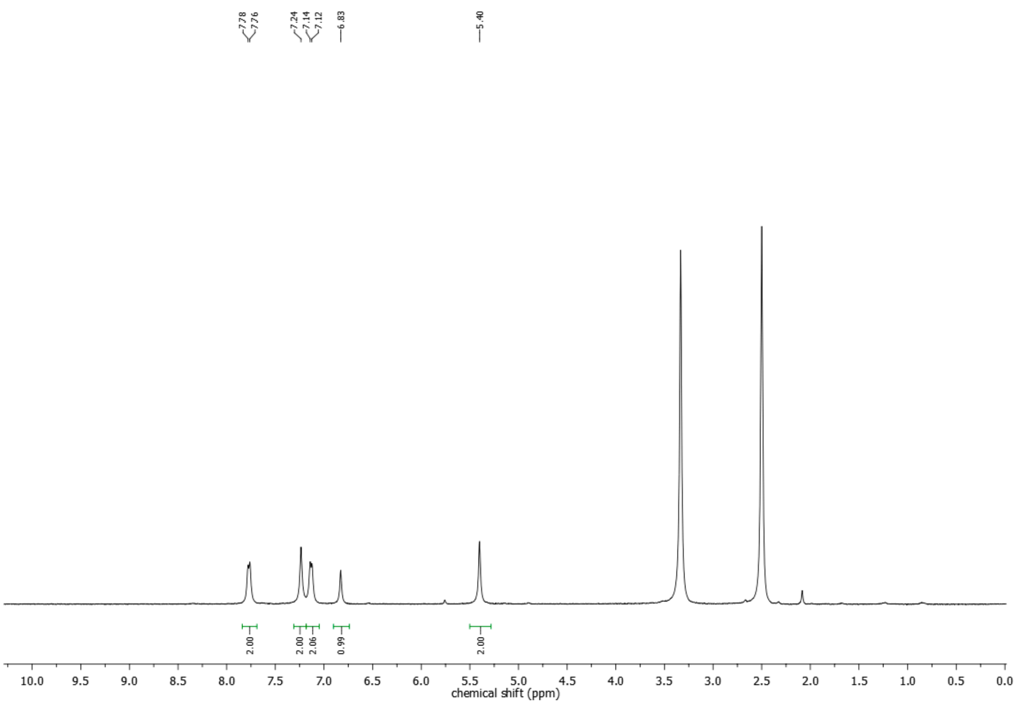

^{1}H NMR compound 2b

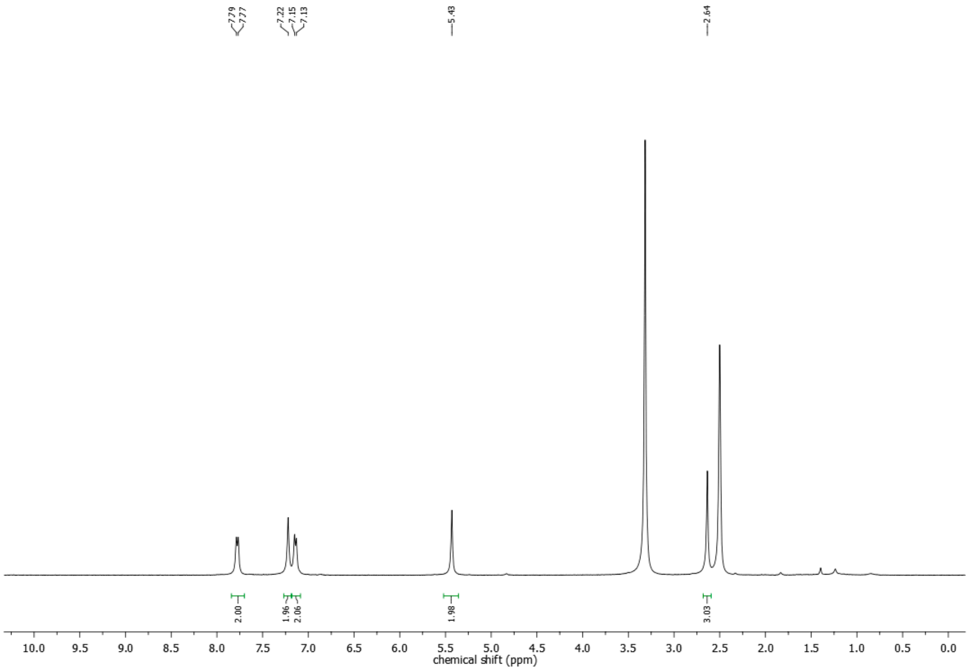

^{13}C NMR compound 2b

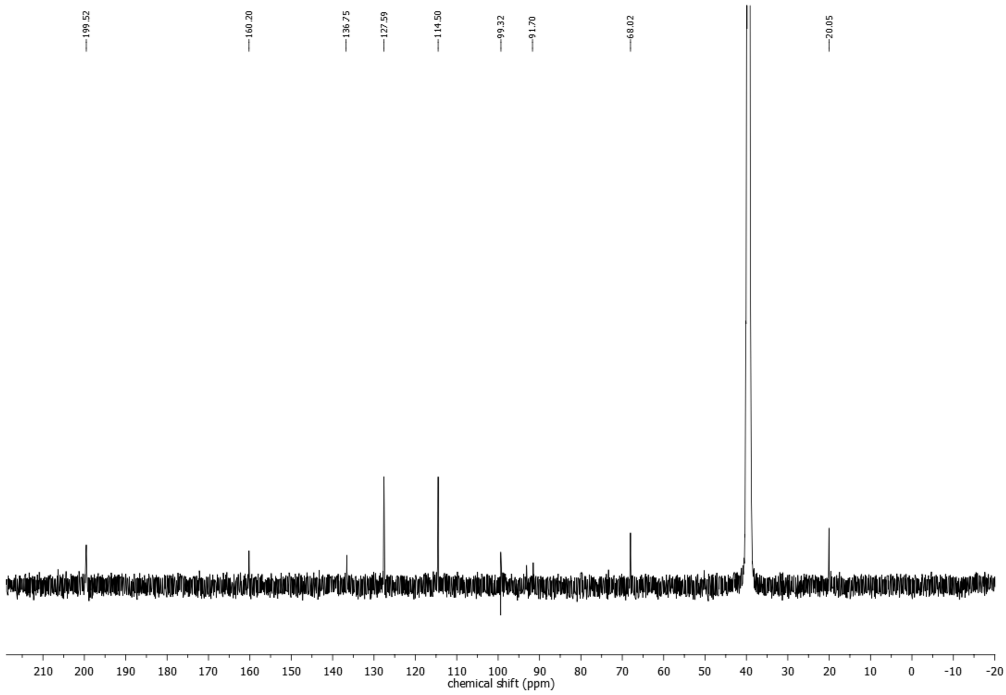

^{1}H NMR compound 3b

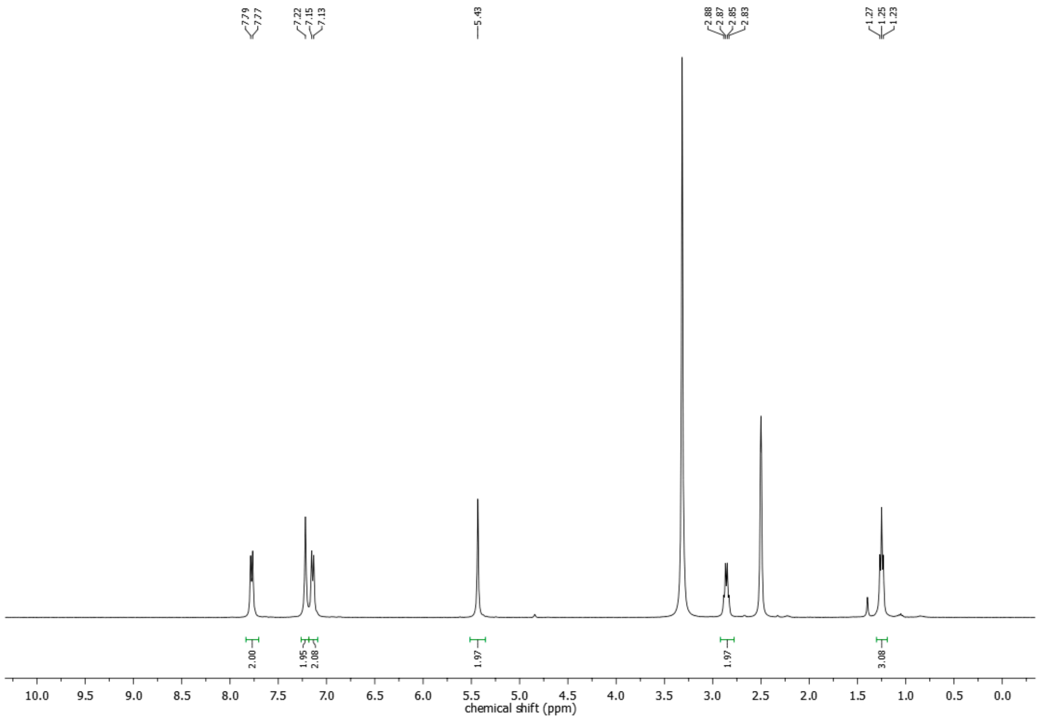

^{13}C NMR compound 3b

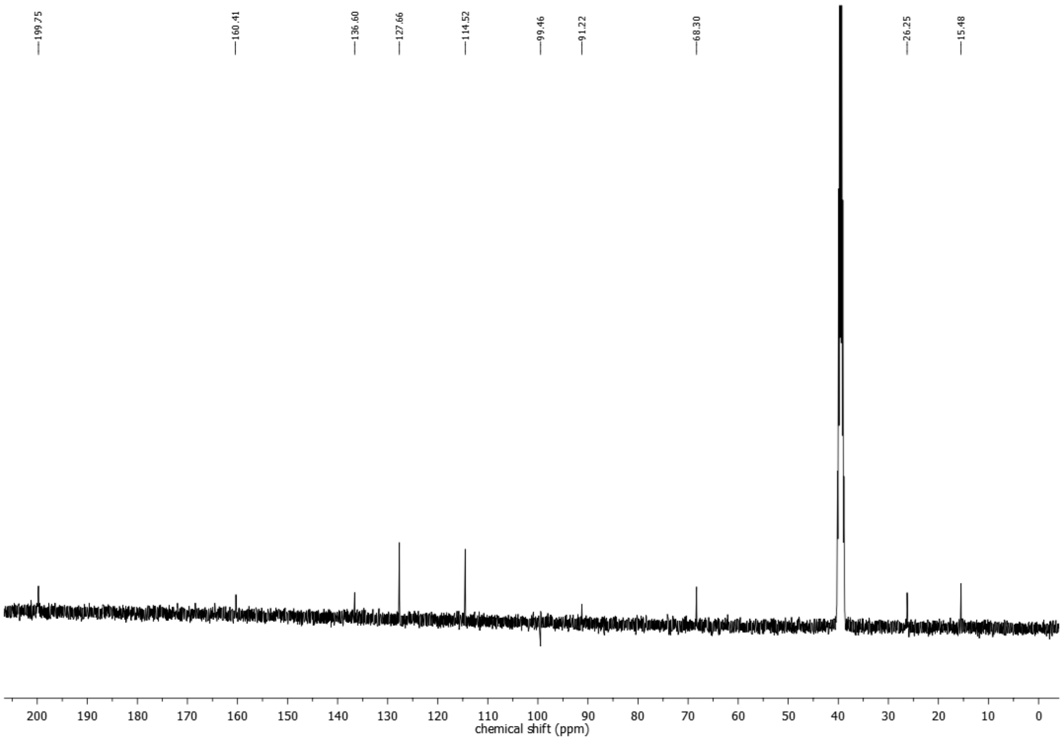

^{1}H NMR compound 1c

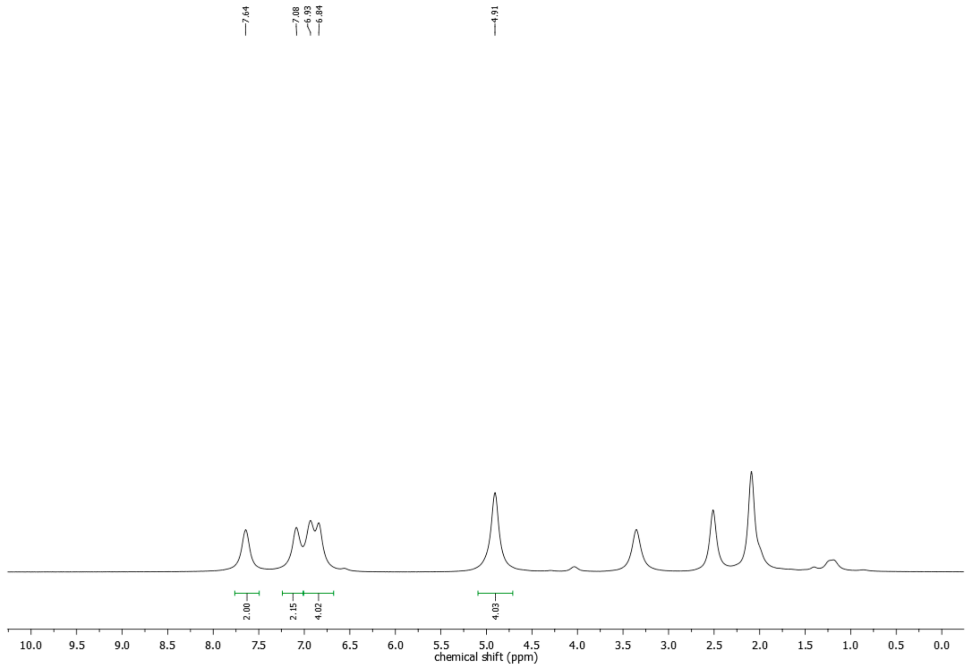

^{1}H NMR compound 2c

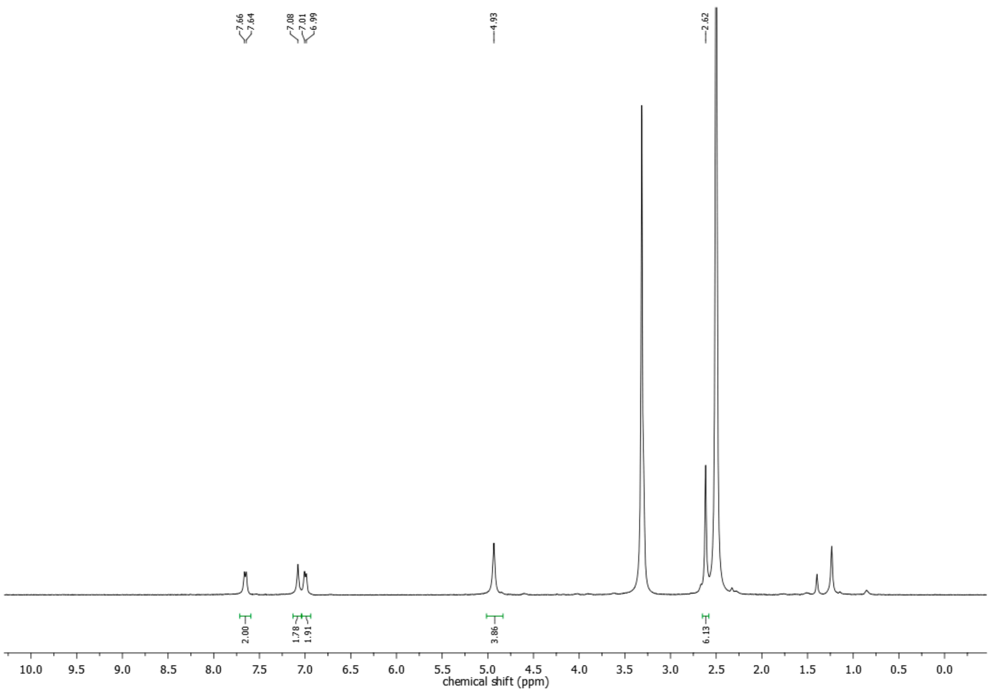

^{13}C NMR compound 2c

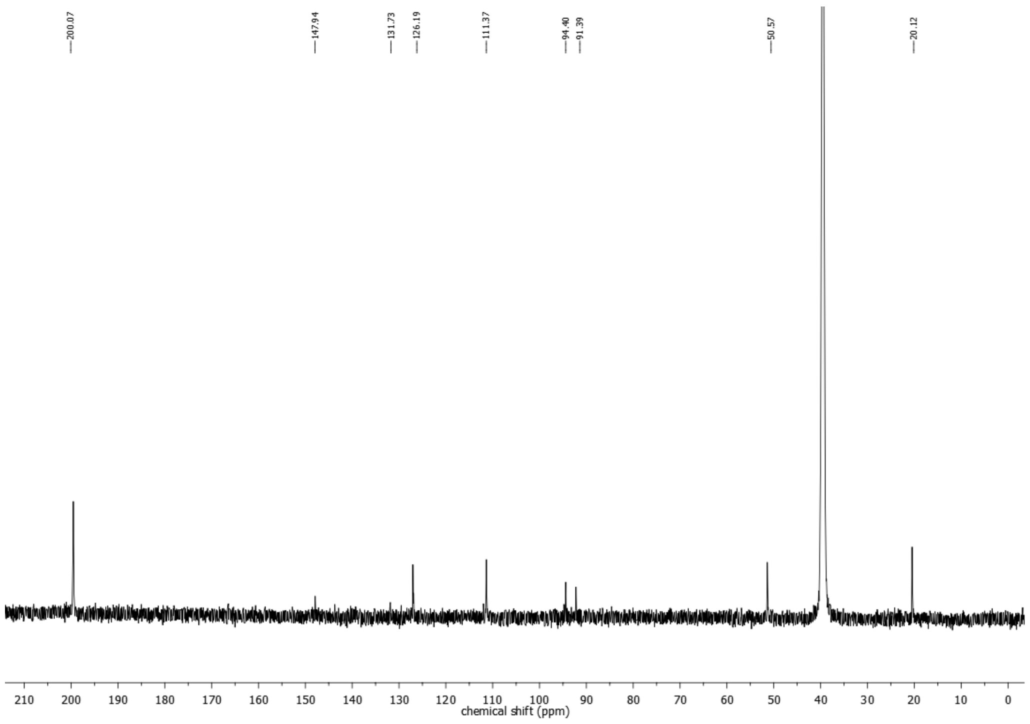

^{1}H NMR compound 3c

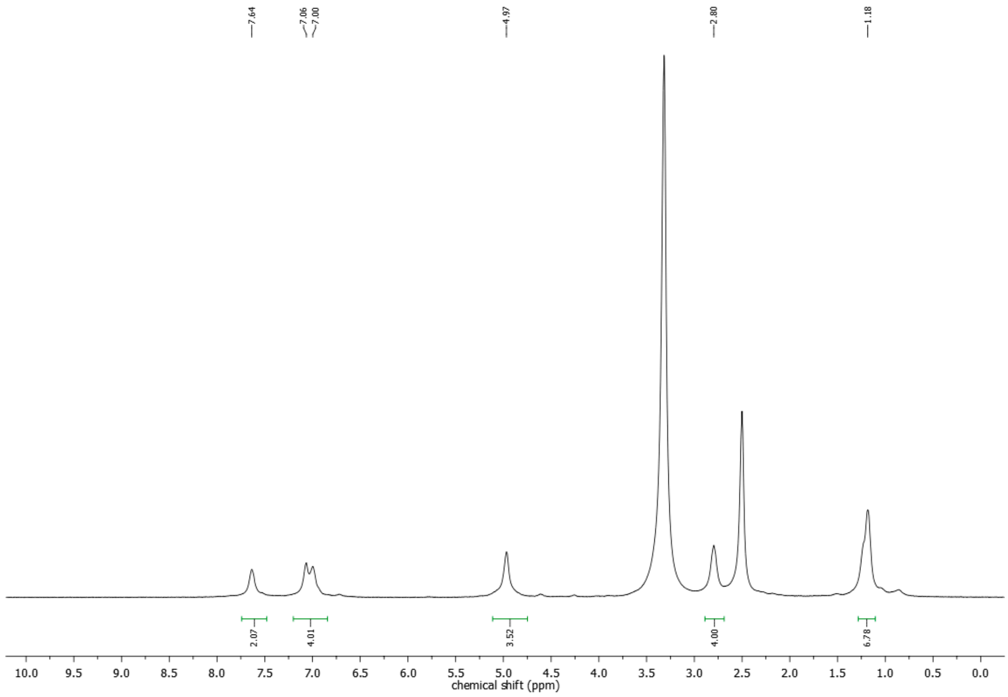

^{13}C NMR compound 3c

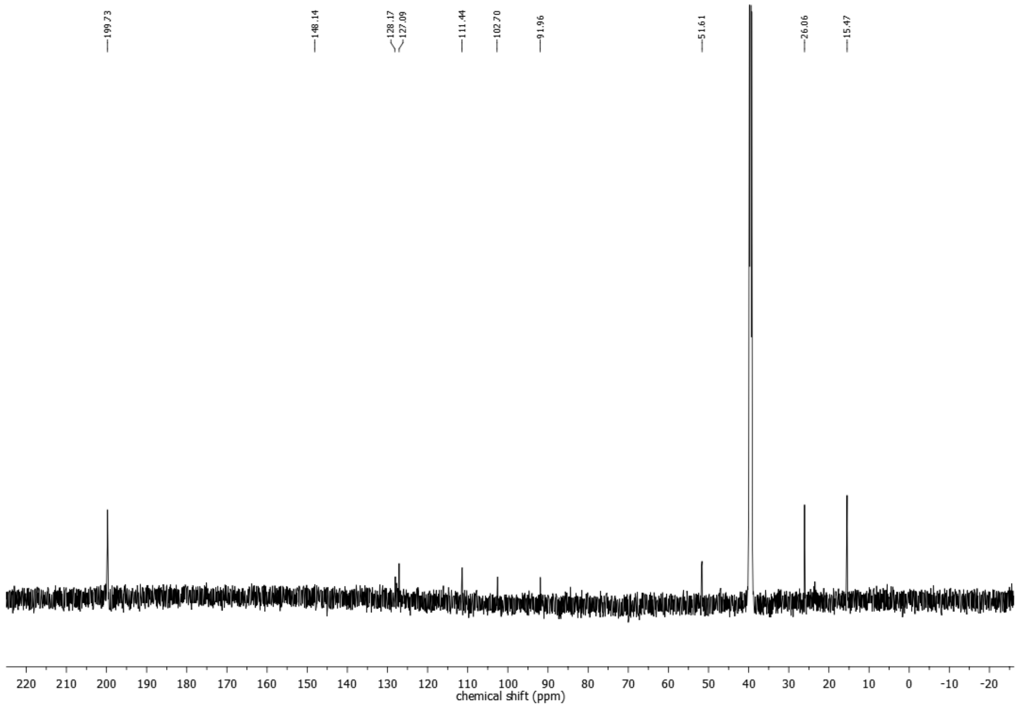

^{1}H NMR compound CO-A

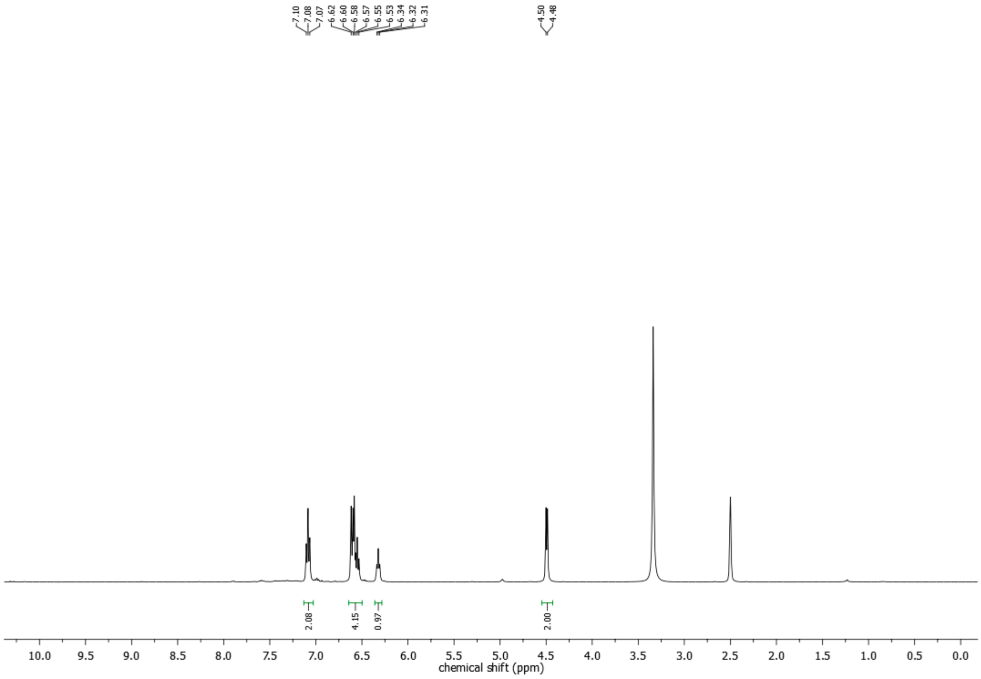

^{13}C NMR compound CO-A

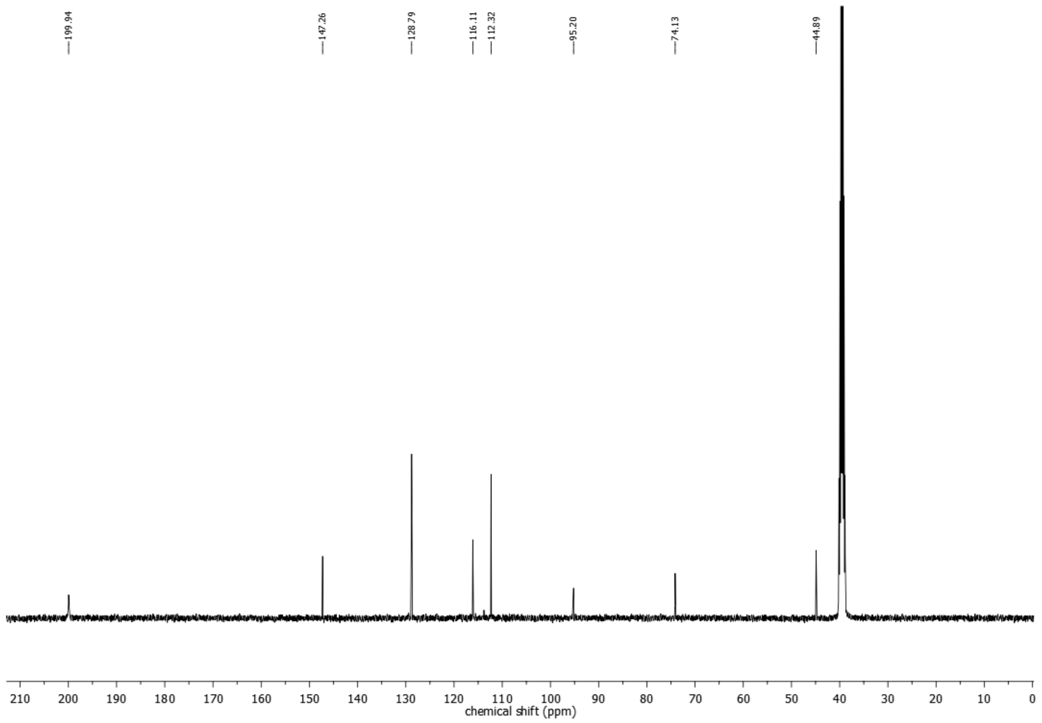

^{1}H NMR compound CO-B

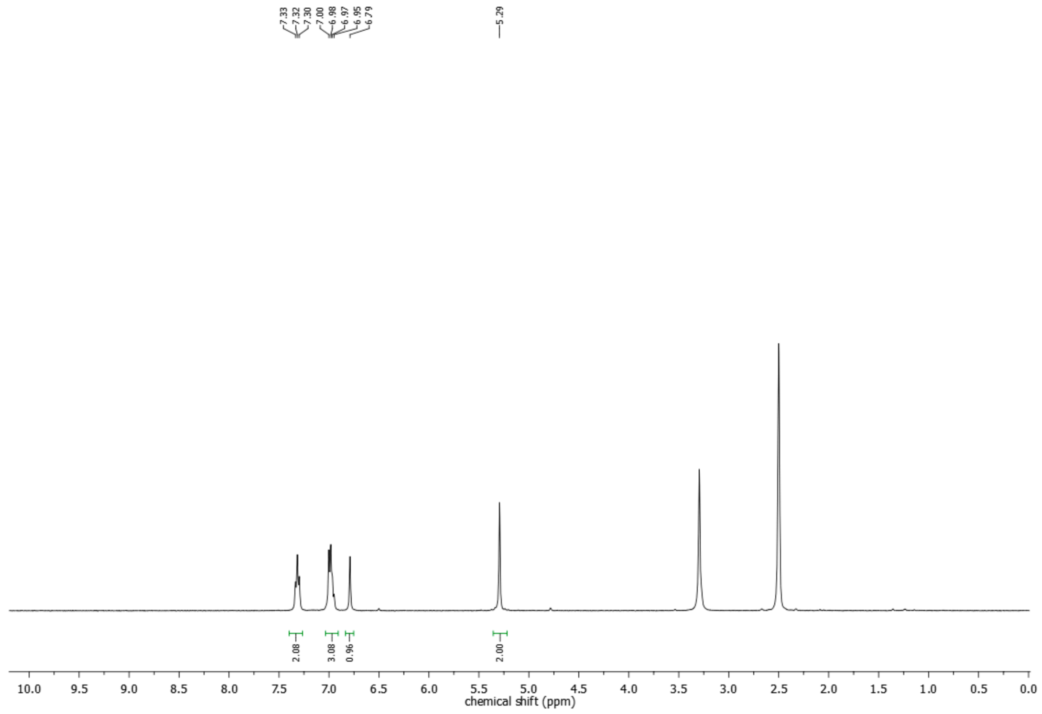

^{1}H NMR compound CO-C

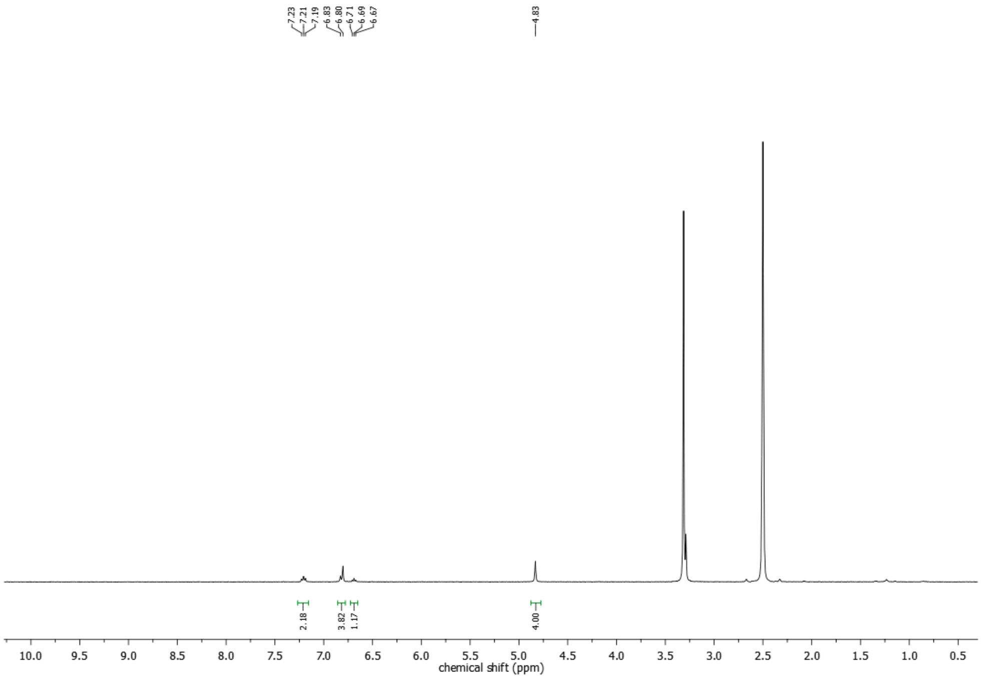

References

1 E. Berrino, L. Milazzo, L. Micheli, D. Vullo, A. Angeli, M. Bozdag, A. Nocentini, M. Menicatti, G. Bartolucci, L. Di Cesare Mannelli, C. Ghelardini, C. T. Supuran and F. Carta, *J Med Chem*, 2019, **62**, 7233–7249.

2 E. Berrino, A. Angeli, D. D. Zhdanov, A. P. Kiryukhina, A. Milaneschi, A. De Luca, M. Bozdag, S. Carradori, S. Selleri, G. Bartolucci, T. S. Peat, M. Ferraroni, C. T. Supuran and F. Carta, *J Med Chem*, 2020, **63**, 7392–7409.

3 S. D. Schimler, D. J. Hall and S. L. Debbert, *J Inorg Biochem*, 2013, **119**, 28–37.
